# An Fc-Engineered Glycomodified Antibody Supports Proinflammatory Activation of Immune Effector Cells and Restricts Progression of Breast Cancer

**DOI:** 10.1158/0008-5472.CAN-24-3174

**Published:** 2025-10-23

**Authors:** Alicia M. Chenoweth, Anthony Cheung, Jitesh Chauhan, Rebecca Adams, Gabriel Osborn, Katie Stoker, Melanie Grandits, Roman Laddach, Jennifer Trendell, Blanca Navarro-Llinas, Erin Suriawinata, Amanda Gross, Amanda Clarke, Lev Brown, Judit Cserny, Lenny Moise, Shashi Jatiani, Alexandra McCraw, Benjamina Esapa, Syed Haider, Jelmar Quist, Kristina M. Ilieva, Anna M. Davies, Pablo Romero-Clavijo, Thomas Sénard, Jack Cheeseman, Richard A. Gardner, Daniel I.R. Spencer, Sophia Tsoka, Sheeba Irshad, James M. McDonnell, Anita Grigoriadis, Andrew N.J. Tutt, Sophia N. Karagiannis

**Affiliations:** 1 Breast Cancer Now Research Unit, School of Cancer & Pharmaceutical Sciences, King’s College London, Guy’s Cancer Centre, London, United Kingdom.; 2 St. John’s Institute of Dermatology, School of Basic & Medical Biosciences & KHP Centre for Translational Medicine, King’s College London, Guy’s Hospital, London, United Kingdom.; 3 Department of Informatics, Faculty of Natural, Mathematical & Engineering Sciences, King’s College London, London, United Kingdom.; 4 SeromYx Systems, Inc., Woburn, Massachusetts.; 5 The Breast Cancer Now Toby Robins Research Centre, The Institute of Cancer Research, London, United Kingdom.; 6 Cancer Bioinformatics, School of Cancer & Pharmaceutical Sciences, King’s College London, Guy’s Cancer Centre, London, United Kingdom.; 7 Randall Centre for Cell and Molecular Biophysics, School of Basic & Medical Biosciences, King’s College London, London, United Kingdom.; 8 Ludger, Ltd., Abingdon, Oxfordshire, United Kingdom.; 9 School of Cancer and Pharmaceutical Sciences, King’s College London, Comprehensive Cancer Centre, Guy’s Hospital, London, United Kingdom.

## Abstract

Fc engineering to enhance antibody effector functions harbors the potential to improve therapeutic effects. Understanding FcγR expression and distribution in the tumor microenvironment prior to and following treatment may help guide immune-engaging antibody design and patient stratification. In this study, we investigated FcR-expressing immune effector cells in HER2^+^ and triple-negative breast cancers (TNBC), including neoadjuvant chemotherapy–resistant disease. FcγRIIIa expression, FcγRIIIa^+^ NK cells, and classically activated (M1-like) macrophages correlated with improved anti-HER2 antibody efficacy. FcγRIIIa protein and FcγRIIIa^+^ NK cells and macrophages were present in primary TNBC and retained in treatment-resistant tumors. FcγRIIIa was spatially associated with folate receptor alpha–positive (FRα^+^) tumor areas at baseline and in residual tumors following neoadjuvant chemotherapy. Wild-type and Fc-engineered antibodies recognizing two breast cancer–associated antigens, HER2 and the emerging TNBC target FRα, were designed and generated to have increased FcγRIIIa-expressing effector cell engagement. The combination of glycoengineering, including fucose removal from the N-linked Fc glycan, and Fc point mutations greatly increased antibody affinity for and retention on FcγRIIIa. The Fc-engineered antibodies enhanced immune effector activity against HER2^+^ breast cancer and TNBC, altering proinflammatory cytokine production by NK cells and tumor-conditioned macrophages and skewing macrophages toward proinflammatory states. Furthermore, the Fc-engineered antibodies restricted orthotopic HER2^+^ and FRα^+^ breast cancer xenograft growth at doses suboptimal for equivalent wild-type antibodies and recruited FcγRIIIa-expressing cells into tumors. Antibody design through combined glycoengineering and Fc point mutations to enhance FcγRIIIa engagement of tumor-infiltrating effector cells may be a promising strategy for developing therapies for patients with aggressive and treatment-resistant breast cancers.

**Significance:**

Assessment of Fc receptors and immune cells in breast cancer enables development of tailored engineering strategies for tumor-targeting monoclonal antibodies with enhanced immune-stimulating and anticancer attributes by combining glycoengineering and Fc mutations.

## Introduction

Breast cancer is the most commonly diagnosed malignancy and the fourth leading cause of cancer deaths worldwide (1). Treatment is often based on the expression of the estrogen and progesterone receptors and HER2. Overexpression of HER2 in breast cancers correlates with poor prognosis and faster relapse (2); however, trastuzumab, an anti-HER2 antibody, has improved patient survival. Despite this, up to 70% of patients with HER2+ breast cancer can become resistant to trastuzumab within a year (3), necessitating new treatments for trastuzumab-resistant and metastatic disease. Triple-negative breast cancers (TNBC), lacking expression of the estrogen receptor, progesterone receptor, or HER2, have a high risk of tumor recurrence and poor prognosis. Recent advances using an antibody–drug conjugate targeting Trop-2 have shown benefits for some patients with TNBC (4); however, novel therapies are needed for those who do not respond or become resistant to currently approved therapies. We previously identified folate receptor alpha (FRα) as a promising target for antibody therapeutics against TNBC, including postchemotherapy residual disease (5).

The tumor microenvironment (TME) can play a considerable role in progression and response to therapeutics (6–8). Immune cells, such as NK cells, T cells, and tumoricidal (M1-like) macrophages, can directly kill cancer cells or stimulate antitumor immune responses, whereas other cells, such as alternatively activated (M2-like) macrophages, can suppress the immune response and aid tumor growth. Thus, the development of novel therapies that not only directly target tumor cells but also skew the immune TME toward a proinflammatory/antitumor state may improve clinical outcomes.

mAbs targeting tumor antigens, such as trastuzumab, can induce Fc-mediated antitumoral effector functions [antibody-dependent cell-mediated cytotoxicity (ADCC) and phagocytosis (ADCP); refs. 9, 10]. These effector functions occur when the Fc region of the IgG antibody binds to cognate FcγRs expressed on the surface of immune effector cells. Human effector cells can express multiple activating FcγRs (FcγRI, FcγRIIa, FcγRIIIa) and the inhibitory FcγRIIb. However, the expression and distribution of FcγRs in the breast cancer TME and their association with clinical outcomes are important unknowns. This knowledge may be critical for the design of Fc-optimized antibodies to enhance immune cell activation and improve therapeutic responses.

Although the majority of clinically available therapeutic antibodies that directly target tumor antigens have an unmodified [wild-type (WT)] IgG1 backbone, engineering the Fc domain either by point mutation or by glycoengineering can improve antitumor effects (11, 12). Margetuximab, featuring the same variable region as trastuzumab, is an Fc-engineered mAb with five amino acid modifications (L^235^V, F^243^L, R^292^P, Y^300^L, and P^396^L), designed with increased affinity to FcγRIIIa and decreased affinity for the inhibitory FcγRIIb (11). Margetuximab showed improved progression-free survival (PFS) in HER2+ patients, demonstrating that improvement in clinical response may be achieved through Fc modification for greater immune activation (13). Different Fc-enhancing modifications such as afucosylation, S^239^D/I^332^E, and S^239^A/E^333^A/K^334^A have also been used with success in FDA-approved antibodies or those in late-stage clinical trials for other types of cancers, but none have been approved for use or evaluated in late-stage clinical trials for breast cancer (data obtained from The Antibody Society: https://www.antibodysociety.org/antibody-therapeutics-product-data/).

Antibody engineering strategies often do not consider the immune context of the TME, including in treatment-resistant tumors, in which there is a significant unmet clinical need. Hence, in this study, we evaluate the presence of FcγRs and FcγR-expressing immune cells in the breast cancer TME, including that of chemotherapy-resistant residual tumors, in which there is a significant unmet need for effective therapies, and baseline tumors from patients who subsequently responded to trastuzumab therapy. Guided by these analyses, we designed triple Fc-engineered mAbs targeting HER2 or FRα, combining both glycoengineering and point mutations to enhance FcγRIIIa affinity. We evaluated antibody affinity for FcγRs, including both FcγRIIIa polymorphic alleles (FcγRIIIa-F^158^ and FcγRIIIa-V^158^). With this, we aim to address the unmet need for the treatment of breast cancer patients with the lower-affinity allele, FcγRIIIa-F^158^, as this group has a poorer response to trastuzumab due to lower binding of IgG1 and thus a poorer ability to mount NK cell–mediated ADCC and other effector functions (14). We assessed these antibodies’ ability to activate NK cells, induce proinflammatory cytokine production, and shift macrophages toward a proinflammatory state. Our engineered antibodies were tested *in vivo* for their ability to restrict tumor growth and recruit FcγRIIIa-expressing cells in HER2+ and FRα+ human breast cancer models.

## Materials and Methods

### Gene expression data from human breast cancer cohorts

Analyses were performed on published bulk RNA sequencing (RNA-seq) or NanoString gene expression [GSE76360, GSE109710, Guy’s cohort of TNBC (15), KCH cohort, NKI cohort] and single-cell RNA-seq (scRNA-seq) gene expression datasets (GSE176078, GSE169246, Bassez cohort, ref. 16). All analyses were performed in RStudio (RRID:SCR_000432). Datasets were downloaded from NCBI GEO DataSets either manually from the website (https://www.ncbi.nlm.nih.gov/gds) or using the R program GEOquery (RRID:SCR_000146). For the bulk RNA-seq gene expression datasets, data were downloaded in the form of processed transcripts per million. Graphs were generated using the ggplot2 package. Tumor immune cell abundance was estimated using the ConsensusTME deconvolution package. scRNA-seq datasets were log-normalized to 10,000 counts per cell and analyzed using the R package Seurat (RRID:SCR_007322). For the GSE176078 dataset, data were stratified into HER2+ and TNBC based on classification by the authors. Only immune cells were selected, and then variable genes were selected by variance stabilizing transformation, followed by scaling and principal component analysis. Unsupervised clustering was performed and visualized by Uniform Manifold Approximation and Projection. Immune cells were manually annotated based on ScType annotation (https://sctype.app) and by checking the expression of key genes. Spatial transcriptomic analyses (from publicly available GSE210616) were performed as previously described to determine the gene expression of *FOLR1* and *FcR* genes per “spot” (17). All datasets interrogated in this study are detailed in Supplementary Table S1.

### Antibody cloning and production

The variable regions of trastuzumab and the FRα-specific antibody MOv18, generated in a human IgG1 isotype format, were cloned into the pVITRO1-hygro-mcs vector as previously described (18). Subsequently, single amino acid mutations were introduced into the WT IgG1 heavy chain sequence using polymerase incomplete primer extension (PIPE) PCR, as previously described (19). Briefly, PIPE primers were designed with the desired point mutations, and PIPE PCR was run using the WT IgG1 pVITRO1 vector as a template to generate linear fragments of the construct containing the mutations with 5’ PIPE overhangs. The primer sequences and cycling conditions are listed in Supplementary Table S2. The PCRs were performed on a ProFlex 3 × 32-well PCR System thermal cycler (Thermo Fisher Scientific). The PCR products were digested with DpnI (New England Biolabs) for 2 hours at 37°C to remove the template. Following digestion, the PCR products were mixed in equal ratios and incubated overnight at room temperature. According to the manufacturer’s instructions, 1.5 µL of this mixture was transformed into One Shot TOP10 chemically competent *Escherichia coli* cells (Thermo Fisher Scientific). Plasmids were extracted using a Midiprep kit (GeneJET), and the sequence was confirmed using Sanger sequencing (Source BioScience Sanger Sequencing Service).

Plasmids were transfected into Expi293F cells (RRID:CVCL_D615) using the ExpiFectamine 293 Transfection Kit (Thermo Fisher Scientific), according to the manufacturer’s instructions. Kifunensine (2 μg/mL) was added to generate glycoengineered antibodies (20). Supernatants of transfected Expi293F cells were harvested after 5 days, centrifuged at 3,000 × *g* for 10 minutes, and filtered using a 0.2 µm membrane. Antibodies were purified using 1 mL Pierce Protein A Columns (Thermo Fisher Scientific) and eluted using glycine (pH 3.5), then neutralized with 1 mol/L Tris (pH 9). Purified antibodies were concentrated and dialyzed into PBS using Amicon Ultra-15 Centrifugal Filters (50 kDa). Antibodies were further purified by high-performance liquid chromatography-size exclusion chromatography (HPLC-SEC) using an S200 Superdex 200 10/300 GL column (Cytiva). Proteins were eluted in PBS (pH 7.4) and 0.1% sodium azide, and fractions containing monomeric material were pooled for further use.

### Cell lines and culture

SK-BR-3 (RRID:CVCL_0033), HCC1954 (RRID:CVCL_1259), JIMT-1 (RRID:CVCL_2077), CAL51 (RRID:CVCL_1110), and T47D (RRID:CVCL_0553) cells were obtained from King’s College London (KCL) Breast Cancer Now Unit between 2019 and 2024 and cultured at 37°C and 5% CO_2_ in either complete DMEM or complete RPMI. All cell lines were authenticated by short tandem repeat profiling. Mycoplasma testing was conducted every 3 months, and cells were used for up to 30 passages.

### Human sample collection

This study was conducted at KCL. Human blood was obtained from the UK National Health Service Blood and Transplant system using anonymous donor leukocyte cones following the provision of written informed consent, in accordance with the Declaration of Helsinki. The study design was approved by London-Chelsea Research Ethics (REC number: 13/LO/1248, IRAS ID 131133).

### Human NK cell isolation

Human NK cells were obtained using RosetteSep Human NK Cell Enrichment Cocktail (STEMCELL Technologies), according to the manufacturer’s instructions. Briefly, blood was diluted with PBS and incubated with the NK Cell Enrichment Cocktail for 30 minutes. Blood was layered over Ficoll-Paque Plus and centrifuged at 600 × *g* without brake for 30 minutes at room temperature. NK cells were harvested using a Pasteur pipette, washed with PBS, and incubated with Red Blood Cell Lysis Buffer for 5 minutes at room temperature. Cells were washed with PBS and then resuspended at 1 × 10^6^ cells/mL in RPMI + 10% FCS + penicillin/streptomycin and cultured overnight before use.

### *Ex vivo* culture of human macrophages

Peripheral blood mononuclear cells (PBMC) were isolated from human volunteer blood diluted with PBS by layering over Ficoll-Paque Plus and centrifuging for 30 minutes at 600 × *g* with no brake at room temperature. PBMCs were harvested using a Pasteur pipette, washed with PBS, and incubated with Red Blood Cell Lysis Buffer for 5 minutes at room temperature. Cells were washed with PBS and plated at 2 × 10^6^ cells/mL in a six-well plate in complete RPMI supplemented with 25 ng/mL M-CSF. The media were replaced 2 hours later with complete RPMI supplemented with 50 ng/mL M-CSF. Cells were incubated for 3 days, and then half the media were replenished with fresh complete RPMI + 50 ng/mL M-CSF before incubation for another 3 days. Macrophages were subsequently detached using PBS + 10 mmol/L EDTA and checked for purity by flow cytometry.

### Cancer cell polarization of macrophages

Breast cancer cells were grown in a T75 flask until around 75% confluent and then cultured for 2 days in serum-free RPMI or DMEM. The supernatant was harvested, filtered through a 0.2 µmol/L syringe filter, and concentrated using an Amicon Ultra-15 Centrifugal Filter Unit (3 kDa). On day 4 of macrophage culture, on the same day that the media are replaced, 35 ng of protein from the cancer cell supernatant is added to the macrophages. Macrophages are cultured for a further 3 days as standard and detached using PBS + 10 mmol/L EDTA for use.

### Surface plasmon resonance

Surface plasmon resonance (SPR) binding experiments were performed using a Biacore T200 instrument (GE Healthcare, RRID:SCR_019718). Anti-His tag antibodies were immobilized onto a CM5 sensor chip using an amine coupling protocol according to the manufacturer’s instructions. His-tagged recombinant FcγR proteins (Sino Biological) were injected at a flow rate of 10 μL/minute for 300 seconds. For binding studies, antibodies in a 2-fold dilution series (0.7–100 nmol/L for FcγRI and FcγRIIIa and 75–1,000 nmol/L for FcγRIIa and FcγRIIb) were injected at a flow rate of 20 μL/minute for 240 seconds, followed by a dissociation time of 900 seconds. All binding experiments were performed at 25°C in 20 mmol/L HEPES (pH 7.4), 150 mmol/L NaCl, and 0.005% (v/v) surfactant P20. BIAevaluation (GE Healthcare, RRID:SCR_015936), Origin 8 (OriginLab, RRID:SCR_014212), and GraphPad Prism (RRID:SCR_002798) were used to analyze and present the data. K_D_ values (nmol/L) of the variant antibodies to the FcRs were calculated from the SPR curves.

### Antigen-specific FcRn binding analysis

An Fc receptor array was employed, which uses fluorescently coded microspheres to capture up to 500 antigen specificities simultaneously and profile the effector capacity of each antigen specificity by determining the ability of these antigen-specific antibodies to interact with Fc receptors. Recombinant human HER2 protein (Sino Biological, cat. #10004-H08H) or recombinant human FOLR1a (FRα) protein (Sino Biological, cat. #11241-H08H) was covalently coupled to Luminex MagPlex carboxylate-modified beads (Luminex/Diasorin S.p.A., cat. #MC10005-01, MC10027-01, MC10033-01, MC10045-01, MC10097-01) using the carbodiimide reagent EDC (Thermo Fisher Scientific, cat. #35391) and amine-reactive Sulfo-NHS ester (Thermo Fisher Scientific, cat. #PIA39269). Coupled beads were diluted to a concentration of 100 microspheres/μL and incubated with the test human monoclonal antibodies and diluted in PBS at a range of 2.3 ng/mL to 5 μg/mL and incubated at room temperature for 2 hours on an orbital mixer, shaking at 800 rpm. The bound antigen-specific antibodies were subsequently stained with PE-labeled tetramerized recombinant FcRn (Duke Protein Production Facility) in a buffered solution with a pH value of either 6.0 or 7.4 for 1 hour at room temperature, shaking at 800 rpm. Fluorescence was analyzed using a Stratedigm S1000EON flow cytometer. The data were reported as the average median fluorescence intensity of PE for a specific bead channel.

### Glycoanalysis via hydrophilic interaction liquid chromatography ultrahigh-performance liquid chromatography

Samples were used as supplied with no cleanup and dried down before use. Samples were treated with PNGase F (New England Biolabs) for N-glycan release and then cleaned up prior to procainamide labeling. Following labeling, samples were cleaned up further to remove excess reagents, eluted in water from the cleanup plate, and concentrated prior to ultrahigh-performance liquid chromatography (UHPLC) fluorescence detection mass spectrometry (MS) analysis as described previously (21). Samples were separated and analyzed via hydrophilic interaction liquid chromatography HPLC using a Thermo Scientific Vanquish UHPLC instrument fitted with a BEH-Glycan 1.7 μm, 2.1 × 150 mm column (Waters) with a fluorescence detector (λex = 310 nm, λem = 370 nm) controlled by Thermo Scientific Xcalibur software (RRID:SCR_014593). MS analysis was performed using an Orbitrap Exploris 120 mass spectrometer (Thermo Fisher Scientific), which was coupled directly after the UHPLC fluorescence detection without splitting but with a pressure relief valve to avoid damage to the fluorometer flow cell. HPLC-electrospray ionization-MS chromatogram analysis was performed using Xcalibur Data Acquisition and Interpretation Software version 4.3 and GlycoWorkbench software (RRID:SCR_000782). Peak integration was performed using the parameterless peak detection (PPD) algorithm on Xcalibur Data Acquisition and Interpretation Software version 4.3, with manual peak integration used when necessary.

### SDS-PAGE electrophoresis

Antibody purification was confirmed using SDS-PAGE. Five micrograms of antibodies in PBS were mixed with 4× Laemmli buffer (nonreducing) or 4× Laemmli buffer + 50 mmol/L DTT (reducing) and then boiled at 95°C for 5 minutes. Twenty microliters was loaded into Mini-PROTEAN TGX Gels (10-well, 30 μL), and gels were run at 150 V for 30 to 45 minutes. Protein was visualized by incubating with InstantBlue Protein Stain for 30 minutes while shaking.

### Flow cytometric analysis of antibody binding to cancer cells and NK cells

Cancer cells were first detached using Trypsin-EDTA and washed in FACS buffer (PBS + 1% FBS + 1 mmol/L EDTA). Detached cancer cells or NK cells were resuspended in FACS buffer at 2 × 10^6^ cells/mL. One hundred microliters of cells were plated per well in a round-bottom 96-well plate and incubated with titrating concentrations of antibody on ice for 1 hour. Cells were washed with FACS buffer and then incubated with 4 μg/mL anti-F(ab′)_2_-Alexa Fluor 647 (Jackson ImmunoResearch Laboratories, Inc., RRID:AB_2337898) for 1 hour on ice and then washed again and resuspended in 200 μL FACS buffer. Samples were acquired using a Beckman Coulter CytoFLEX, and analysis was performed using FlowJo software.

### Cancer cell proliferation assays

A total of 1,000 cells in 100 μL were plated per well in a flat-bottom 96-well plate in complete RPMI or complete DMEM. Antibodies were serially diluted 10-fold starting from 10 μg/mL and added to the appropriate wells, and the plate was incubated in the Incucyte S3 Zoom system (Sartorius, RRID:SCR_019874) at 37°C and 5% CO_2_. Images were taken every 24 hours for 5 days, and cell density was calculated using the Incucyte analysis software. The level of % cell proliferation was calculated by dividing the calculated cell density by the average cell density of no antibody control conditions.

### NK cell IgG–FcγR cross-linking assays

A total of 400,000 NK cells were plated per well in complete RPMI in a round-bottom 96-well plate and incubated for 10 minutes at 37°C and 5% CO_2_ with 20 μg/mL antibodies. Polyclonal anti-IgG (10 μg/mL; Stratech, RRID:AB_2337534) was added to each well and incubated for a further 30 minutes. Cells were washed with FACS buffer and stained with anti-CD56, anti-CD69 (RRID:AB_2561909), anti-CD107a (RRID:AB_1186036), and anti-CD16 (RRID:AB_492976) in FACS buffer for 1 hour on ice. Cells were washed and resuspended in 200 μL FACS buffer. Samples were acquired using a Beckman Coulter CytoFLEX (RRID:SCR_025067), and analysis was performed using FlowJo software (RRID:SCR_008520).

### NK cell coculture with cancer cells

Cancer cells were detached using Trypsin-EDTA and washed in complete RPMI or DMEM. Fifty microliters of 4 × 10^6^/mL NK cells and 50 μL of 0.4 × 10^6^/mL cancer cells were added to a round-bottom 96-well plate. Antibodies were serially diluted 10-fold starting from 10 μg/mL and added to the appropriate wells in the presence or absence of protease inhibitors, 50 µmol/L GM-6001 or TAPI-2, and plates were incubated at 37°C and 5% CO_2_ for either 4 hours (ADCC assays) or 6 hours (cytokine release assays). For ADCC assays, plates were spun down, and 50 μL of supernatant was added to a new round-bottom 96-well plate along with 50 μL of lactate dehydrogenase assay reagent from the CyQUANT LDH Cytotoxicity Assay Kit (Invitrogen). After 30 minutes of incubation, 50 μL of stop solution was added, and absorbance at 490 and 680 nm was read on a FLUOstar Omega Microplate Reader (BMG Labtech, RRID:SCR_025024). % Max lysis was calculated using a positive control of lysed cancer cells, and EC_50_ was calculated using GraphPad Prism. The cells from these assays were washed with FACS buffer and incubated on ice for 1 hour with anti-CD56 (RRID:AB_314446) and anti-CD16 (RRID:AB_492976) before being washed and resuspended in 200 μL of FACS buffer. Samples were acquired using a Beckman Coulter CytoFLEX, and analysis was performed using FlowJo software. For cytokine release assays, plates were spun down, and the supernatant was retrieved, and ELISA was performed as per the manufacturer’s instructions. Absorbance at 490 nm was measured on a FLUOstar Omega Microplate Reader (BMG Labtech). The standard curve and cytokine concentration were calculated using GraphPad Prism. ADCC assays were also performed at a 3:1 NK cell–to–target cell ratio. Target cells were stained with CellTrace Far Red and incubated for 4 hours with engineered antibodies and NK cells. Cells were subsequently treated with Accutase, then washed and stained with the Zombie Green Fixable Viability Kit (BioLegend), and fixed. Lysis was measured by flow cytometry. Nonspecific lysis, as measured using a negative control mAb, was subtracted to obtain specific lysis values.

### Macrophage cocultures with cancer cells

For ADCC/ADCP flow cytometric assays, 16 hours before coculture, cancer cells were washed in Hank’s Balanced Salt Solution and incubated with 0.5 mmol/L carboxyfluorescein succinimidyl ester (CSFE) for 10 minutes at 37°C and 5% CO_2_. Cells were washed with complete RPMI or DMEM and incubated overnight. Fifty microliters of 1 × 10^6^/mL CSFE-labeled cancer cells and 50 μL of 3 × 10^6^ cells/mL macrophages were added to a round-bottom 96-well plate in the presence of 10 μg/mL antibody and incubated for 3.5 hours. Cells were washed in FACS buffer and incubated for 1 hour on ice with anti-CD14 (RRID:AB_830685). Cells were washed again in FACS buffer and resuspended in DAPI-containing FACS buffer. Samples were acquired using a Beckman Coulter CytoFLEX, and analysis was performed using FlowJo software. ADCC and ADCP were calculated as previously described (22).

For cytokine assays and phenotyping by flow cytometry, 50 μL of 0.4 × 10^6^/mL of unlabeled cancer cells and 50 μL of 2 × 10^6^ cells/mL macrophages (either unpolarized or cancer cell–polarized) were added to a round-bottom 96-well plate and incubated for 16 hours. Plates were spun down, and the supernatant was retrieved, and ELISA was performed as per the manufacturer’s instructions. Absorbance at 490 nm was measured on a FLUOstar Omega Microplate Reader (BMG Labtech). The standard curve and cytokine concentration were calculated using GraphPad Prism. The cells were washed in FACS buffer and incubated for 1 hour on ice with anti-CD14 (RRID:AB_314186), anti-CD80 (RRID:AB_2687024), anti-CD163 (RRID:AB_2563475), and anti-CD16 (RRID:AB_2562085). Cells were washed again in FACS buffer and resuspended in FACS buffer. Samples were acquired using a Beckman Coulter CytoFLEX, and analysis was performed using FlowJo software.

### *In vivo* models

Animals were handled in accordance with Institutional Committees on Animal Welfare [The Home Office Animals (Scientific Procedures) Act, 1986]. All studies were approved by the KCL Biological Services Unit Ethics Committee and conducted under an approved Home Office project license [project licenses (PPL): PP9706060 and PP5797318]. Our study exclusively examined female mice because the disease modeled is predominantly relevant in females.

Six-week-old female NOD.Cg-Prkdc^scid^ II2rg^tm1Wjl^/SzJ (NSG; RRID:IMSR_JAX:005557) mice were used for orthotopic injection of 1 × 10^6^ (25 μL cells in PBS mixed with 25 μL Matrigel) of CAL51 or HCC1954 cells into the mammary fat pad. Mice were randomly assigned to a treatment group after tumor establishment, and the number of animals, concentration, and administration schedule of mAb treatment were based on previous studies (5) and planned in accordance with the reduce and refine guidelines of the Institutional Committees on Animal Welfare [The Home Office Animals (Scientific Procedures) Act, 1986]. Once tumors reached palpable size (3 mm × 3 mm), mice were injected intravenously via the tail vein with 1.5 × 10^6^ purified NK cells or 10 × 10^6^ PBMCs with antibody (1 or 2 mg/kg). Mice were injected intravenously weekly with 1 or 2 mg/kg antibody. For mice reconstituted with human NK cells, 250 ng human IL15 was added with the initial NK cell injection and subsequent antibody injections, as well as 250 ng human IL15 added via i.p. injection weekly, 3 days after antibody injections.

To assess the serum clearance of Fc-engineered antibodies, nontumor-bearing NSG mice (NOD.Cg-*Prkdc*^scid^ Il2rg^tm1Wjl^/SzJ) were intravenously injected with 10 × 10^6^ PBMCs from human volunteers, followed by either WT antibody (2 mg/kg; *n* = 5) or S^239^D and I^332^E/glycomodified (DE/GM) variant antibody (2 mg/kg; *n* = 5). Blood samples (50 µL) were collected from the tail vein at 1, 24, and 72 hours, as well as at 7 and 14 days after injection, using Microvette 100 Serum collection tubes (Sarstedt; 20.1280.100). Samples were centrifuged according to the manufacturer’s instructions to isolate serum and stored at −80°C until analysis. Total human IgG concentrations in serum were measured using the Invitrogen Human IgG (Total) ELISA Kit (Invitrogen; BMS2091), following the manufacturer’s protocol. Prior to analysis, serum samples were diluted 1:50 in the assay buffer provided with the kit.

For the human CD34^+^ hematopoietic stem cells engrafted NOD.Cg-*Prkdc*^scid^ Il2^rgtm1Wjl^ Tg(CMV-IL3,CSF2,KITLG)1Eav Tg(IL15) model, 6-week-old female mice were purchased from The Jackson Laboratory. Blood was collected via the tail vein to confirm human immune cell engraftment by FACS (baseline, day 0). HCC1954 cells (2.5 × 10^6^) were orthotopically injected into the mammary fat pad and randomly assigned to treatment groups after tumor establishment. Mice were treated with 3 mg/kg of antibody or PBS vehicle control once weekly by intravenous injection. Blood was collected again on day 14 from the baseline.

Experiments were terminated once the tumor mean diameter reached >12 mm or a maximum dimension reached 15 mm or if tumors were wet or ulcerated. Kaplan–Meier survival graphs were generated using a “death” cutoff point of 75% of max tumor growth. Where appropriate, tumors were processed into a single-cell suspension, and immune cell infiltration was measured via flow cytometry.

### Power calculations and statistical analyses

For *in vitro* and *in vivo* experiments, sample sizes were based on estimates from power analysis with 90% power and a significance level of 5%.

Graphs were generated, and statistical analysis was performed using GraphPad Prism 10. Means and SEM are shown for *in vitro* and *in vivo* experiments. All statistical tests comparing three or more subgroups were determined using one-way ANOVA. Statistical methods used for each experiment are described in the figure legends. For all figures, *, *P* < 0.05; **, *P* < 0.01; ***, *P* < 0.001; ****, *P* < 0.0001.

## Results

### NK cells and macrophages express FcγRIIIa in HER2+ breast cancer and TNBC, and FcγRIIIa+ NK cell and classically activated macrophage infiltration at baseline are associated with subsequent trastuzumab response

**Figure 1.**
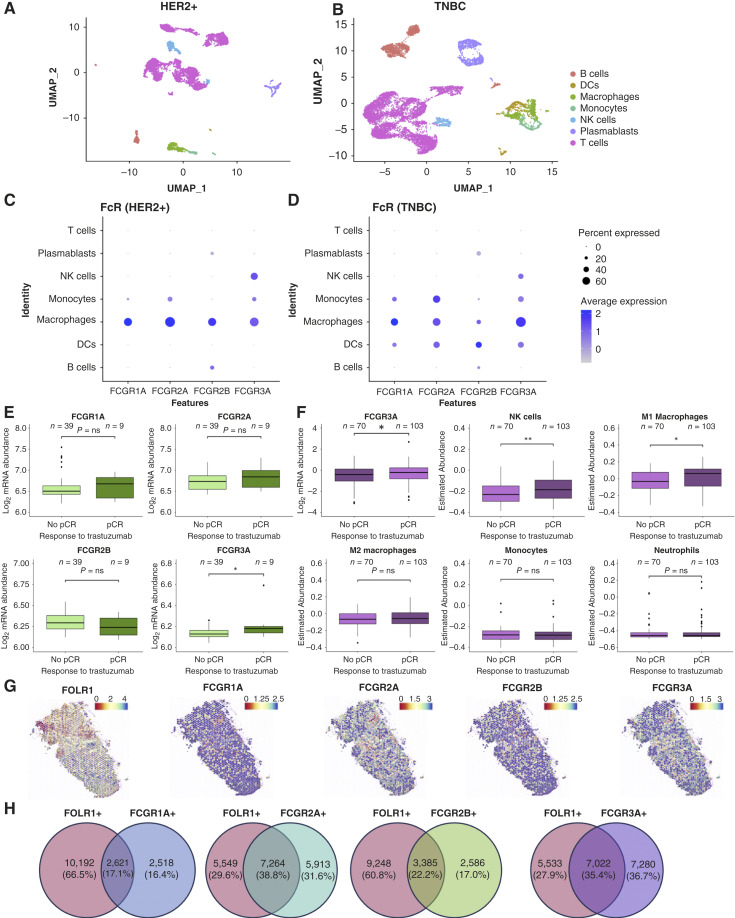
Transcriptomic analysis of FcγR and immune cell expression. **A** and **B,** Uniform Manifold Approximation and Projection (UMAP) visualization of tumor-infiltrating lymphocytes from pretreatment samples, HER2+ (**A**) and TNBC (GSE176078; **B**). **C** and **D,***FCGR *genes were plotted for each immune cell subtype for HER2+ (**C**) and TNBC (**D**). **E,** Comparison of normalized log_2_*FCGR* expression from bulk mRNA data from HER2+ patients before trastuzumab treatment (GSE76360). Patients were classified into pCR or no pCR. Statistical significance was calculated using the Wilcoxon test. **F,***FCGR3A* expression and immune cell subtypes estimated using ConsensusTME from bulk mRNA data from HER2+ patients before trastuzumab treatment (GSE109710). **G,** Representative images of pretreatment TNBC, evaluated by spatial transcriptomic analysis of the publicly available dataset GSE210616 (24 tumor sections from 12 patients). **H,** Venn diagrams of coexpression of *FOLR1* and *FCGR* from spatial transcriptomic analysis. *, *P* < 0.05; **, *P* < 0.01; ns, not significant.

To assess the potential of Fc-active antibodies to engage with FcγRs on immune cells and thus engender effector functions within tumors, we interrogated publicly available scRNA-seq data (GSE176078, ref. 23; Supplementary Table S1). In three primary HER2+ (Fig. 1A) and four TNBC tumors (Fig. 1B), infiltrating T cells, B cells, NK cells, plasmablasts, dendritic cells (DC), monocytes, and macrophages were identified, in line with previous reports (7). Monocytes, macrophages, and NK cells in HER2+ and TNBC all expressed *FCGR3A*, whereas *FCGR3A* was also expressed on DCs in TNBC. In HER2+ breast cancer, *FCGRIA* and *FCGR2A* were expressed on monocytes and macrophages (Fig. 1C), whereas in TNBC, *FCGRIA* and *FCGR2A* were expressed on monocytes, macrophages, and DCs (Fig. 1D). The inhibitory receptor *FCGR2B* was expressed on monocytes, macrophages, plasmablasts, and B cells in both cohorts and was additionally expressed on DCs in TNBC (Fig. 1C and D). These findings suggest that immune cells in the TME express various FcγRs, potentially enabling antibodies to induce effector functions.

We confirmed FcR expression in a bulk RNA-seq dataset of 140 TNBCs (Supplementary Table S1; ref. 15). Most lesions expressed *FCGR3A* and *FCGR2A* as well as the inhibitory *FCGR2B*, whereas only a subset expressed *FCGRIA* (Supplementary Fig. S1). A different small subset of patient tumors had very low expression of all *FCGR *genes, potentially marking these as less likely to respond to antibody therapies in which the mode of action relies on engagement with FcγR-expressing immune cells. This may provide a potential mode of therapy stratification.

To explore conditions favoring antibody-mediated antitumoral effects, we examined pretreatment *FCGR* gene expression and immune cell profiles in publicly available bulk RNA-seq data of HER2+ primary tumors taken prior to trastuzumab treatment (GSE76360, ref. 24; Supplementary Table S1). The cohort was stratified based on whether a pathologic complete response (pCR) was achieved at the end of treatment. *FCGRIA*, *FCGR2A*, and *FCGR2B* expression was comparable between groups (Fig. 1E), whereas baseline *FCGR3A* expression was significantly higher in patients who subsequently achieved pCR compared with those who did not. We confirmed the same correlation between higher *FCGR3A* expression and pCR in another bulk RNA-seq dataset (GSE109710, ref. 25; Supplementary Table 1; Fig. 1F). These findings suggest that FcγRIIIa may be a factor associated *a priori* with clinical response to trastuzumab and are in line with clinical observations that FcγRIIIa polymorphic variants can influence clinical response to trastuzumab (26). In the latter dataset, we also used ConsensusTME (27) to estimate the relative immune effector cell abundance in the TME. Patients who achieved pCR had significantly higher NK cell and classically activated M1-like macrophage infiltration prior to treatment compared with those who did not (Fig. 1F). Conversely, there were no significant differences in the expression of M2 macrophage, monocyte, or neutrophil marker signatures in patients who did versus those who did not achieve pCR. Together, these analyses indicate that the presence of FcγRIIIa, NK cells, and M1-like macrophages in the TME at baseline may be determinants of trastuzumab efficacy.

To determine whether these immune cells harbor the capacity to exert a similar effect in response to an antibody treatment, we interrogated spatial transcriptomic data of 12 treatment-naïve TNBC patients from GSE210616 (Supplementary Table S1; Fig. 1G). Spatial mapping of expression areas of *FOLR1* revealed coexpression with *FCGR3A* in 55.9% of *FOLR1*+ spots, demonstrating the topological proximity of FcγRIIIa-expressing effector cells with FRα-expressing cells (Fig. 1H). Similarly, *FCGR2A* was coexpressed in 56.7% of *FOLR1*+ spots, whereas coexpression of *FCGRIA* and *FCGR2B* was lower at 20.4% and 26.7% of *FOLR1*+ spots, respectively, likely partly due to the lower overall expression of these two receptors. Overall, these data demonstrate prominent expression of FcγRIIIa in treatment-naïve TNBC tumors proximal to the tumor antigen–expressing cells, suggesting a potential therapeutic opportunity for FcγRIIIa-enhanced antibodies.

### FcγRIIIa, FcγRIIIa+ NK cells, and macrophages are retained in treatment-resistant TNBC and are spatially associated with FRα+ tumor areas at baseline and following neoadjuvant therapy

**Figure 2.**
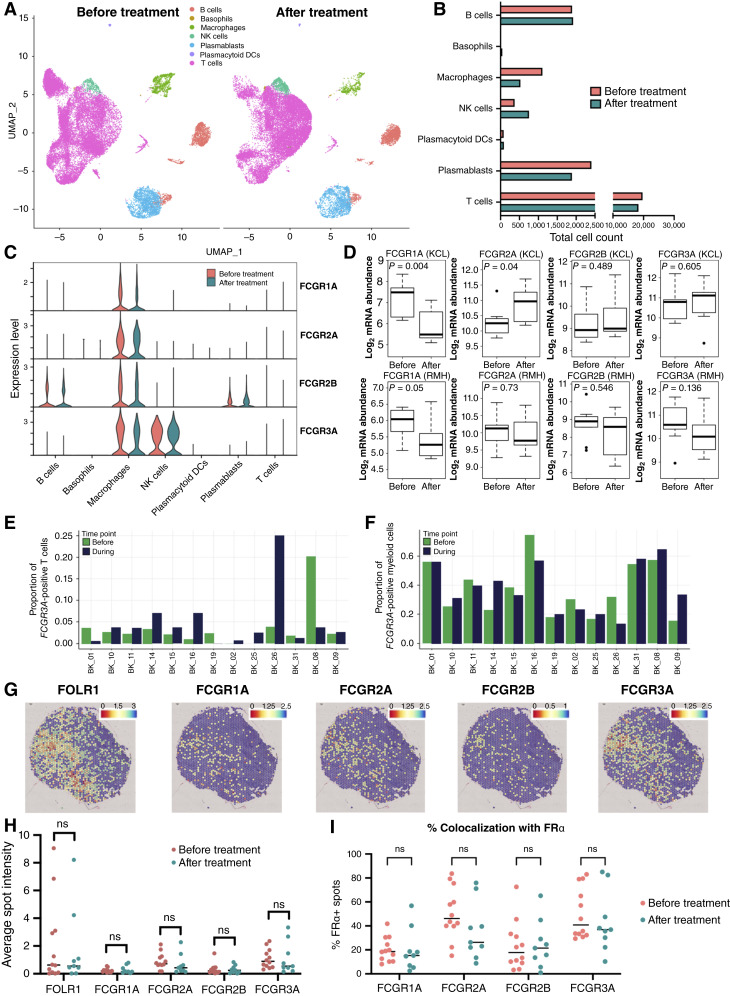
Transcriptomic analysis of *FCGR* and immune cell expression in patients before and after chemotherapy and immunotherapy treatment. **A,** Uniform Manifold Approximation and Projection (UMAP) visualization of tumor-infiltrating lymphocytes in matched tumor samples taken before and after chemotherapy (GSE169246). **B,** Comparison of total immune cell counts of patients before and after chemotherapy treatment (GSE169246). **C,** Normalized log_2_ expression of *FCGR1A*, *FCGR2A*, *FCGR2B*, and *FCGR3A* (GSE169246). **D,** NanoString FcγR gene expression from two TNBC cohorts: Royal Marsden Hospital (RMH) and Guy’s and St Thomas’ NHS Trust (KCL). **E** and **F,** Proportion of *FCGR3A*-positive T cells and NK cells (**E**) and myeloid cells (**F**) determined by scRNA-seq analysis in TNBC patients before and during anti–PD-1 therapy. **G,** Representative spatial transcriptomic analysis from the publicly available dataset GSE210616. **H **and** I,** Spatial transcriptomic analyses of *FOLR1* and *FCGR* expression (**H**) and colocalization frequency of *FCGR* genes with *FOLR1* (**I**) before treatment (*n* = 12) and after treatment (*n* = 9). Statistical significance was determined using two-way ANOVA. ns, not significant.

TNBC patients can receive a variety of therapies, including chemotherapy. We investigated whether FcγR in the TME persisted after paclitaxel chemotherapy using a publicly available dataset (GSE169246; ref. 28; Supplementary Table S1). It revealed a trend toward lower macrophage and higher NK cell densities following neoadjuvant chemotherapy (NAC), consistent with previous reports (Fig. 2A and B; ref. 7). *FCGR* gene expression remained unchanged, with sustained *FCGR3A* expression on macrophages and NK cells after treatment (Fig. 2C). Two TNBC cohorts (KCL and Royal Marsden Hospital, Supplementary Table S1; ref. 7) showed reduced *FCGRIA* but retained *FCGR3A* expression after NAC (Fig. 2D). In a separate TNBC dataset (Bassez cohort, ref. 16; Supplementary Table S1), we found similar proportions of *FCGR3A*+ cells in the T-cell compartment (which contains NK cells) and myeloid cells in tumors before and during anti–PD-1 immunotherapy (Fig. 2E and F).

Spatial transcriptomic analysis (from GSE210616) of nine residual tumors (Fig. 1G; Supplementary Table S1) showed no change in the expression of *FOLR1* or *FCGR* genes compared with the untreated cohort (Fig. 2H). *FCGR2*A and *FCGR3A* remained colocalized with *FOLR1* in chemotherapy/immunotherapy-treated patients at comparable levels to the treatment-naïve group (Fig. 2I). These findings indicate that even after treatment, FcγRs are retained and in close proximity in target antigen-expressing areas and thus have the potential to be engaged by antibody therapeutics.

In summary, scRNA-seq and spatial profiling confirmed immune cell persistence and maintained FcγR expression and colocalization with FRα in the TNBC TME following different chemotherapy and immunotherapy treatments, highlighting opportunities for antibody design to harness FcγRIIIa-engaging immune cell-mediated antitumor functions in treatment-resistant disease.

### Breast cancer–targeting Fc-engineered IgG1 antibodies combining Fc mutation and glycomodification display enhanced affinity for FcγRIIIa compared with WT IgG1

Based on the presence and retention of FcγRIIIa-expressing effector cells in HER2+ breast cancer and TNBC, including residual disease, and their association with clinical outcomes, we hypothesized that Fc-engineering mAbs with enhanced FcγRIIIa affinity could enhance antitumor mechanisms.

We generated Fc-modified antibodies to enhance FcγRIIIa binding attributes on immune cells by a combination of known modifications to the Fc region structure: (i) glycoengineering of the N-linked Fc glycan, including fucose removal (29), and (ii) two mutations in amino acid positions S^239^D and I^332^E of the Fc region (defined as DE; refs. 19, 30). Each modification was previously tested for Fc engagement with cognate receptors, but these have not been previously combined in a full-length engineered antibody. We generated four variants of trastuzumab: IgG1-WT, IgG1-GM, IgG1-DE, and IgG1-DE/GM. We also generated these same Fc variants using the anti-FRα mAb MOv18 to target TNBC (Supplementary Fig. S2). We compared the four anti-HER2 Fc-engineered variants against the only FDA-approved Fc-engineered anti-HER2 margetuximab.

**Figure 3.**
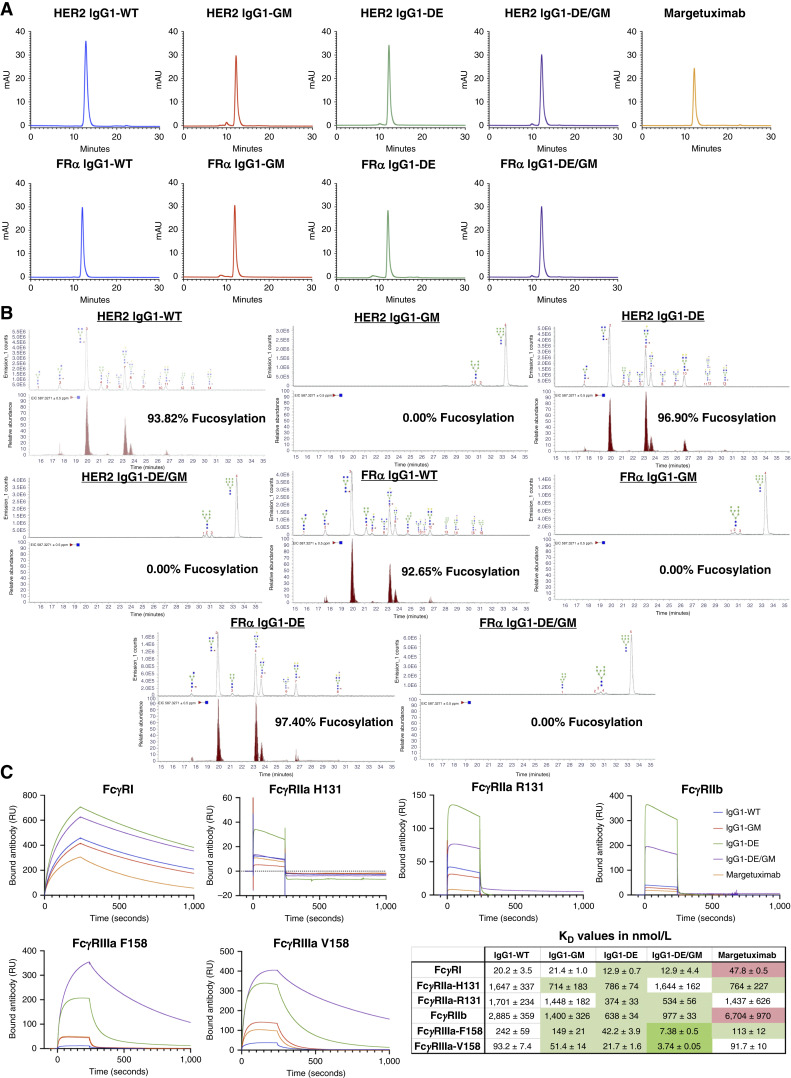
Production and biophysical characterization of FcγRIIIa-enhanced antibodies. **A,** HPLC–size exclusion chromatography chromatograms for the antibody variants. **B,** HPLC–fluorescence detection chromatograms (top) with suggested glycan structures assigned to main peaks based on m/z masses and predicted monosaccharide compositions, with an extracted ion chromatogram (bottom) showing the presence or absence of fucosylated structures. The percentage of fucosylation was calculated by analyzing the number of predicted monosaccharide compositions that contained fucose compared with those predicted not to contain fucose. **C,** SPR analysis of antibody variants binding to human FcγRs. Curves shown are at a concentration of 100 nmol/L (FcγRI and FcγRIIIa) or 1,000 nmol/L (FcγRIIa and FcγRIIb).

All variants were expressed at comparable levels (Supplementary Table S3), intact, and without significant degradation or aggregation (Fig. 3A; Supplementary Fig. S3). We also confirmed that our in-house generated anti-HER2 IgG1-WT was in sequence and functionally identical to commercial trastuzumab, comparing binding to HER2+ cells, binding to FcγRIIIa on NK cells, and the ability to engender NK cell–mediated ADCC of cancer cells (Supplementary Fig. S4).

Glycan analysis via ultra-hydrophilic interaction liquid chromatography HPLC demonstrated the presence of complex glycans on WT and DE variants, with glycan structures containing mannose, GlcNAc, galactose, fucose, and sialic acid residues (Fig. 3B). Contrastingly, the GM and DE/GM antibodies, which were produced in the presence of the mannosidase inhibitor kifunensine, had much less variation in their glycan structures: the majority of glycan structures only contained mannose and GlcNAc residues (Fig. 3B). These glycomodified variants did not contain any galactose or sialic acid residues and, importantly, were also completely afucosylated.

SPR analysis was performed to measure antibody affinity to FcγRI, the two main allelic forms of FcγRIIa (H^131^ and R^131^), the inhibitory FcγRIIb, and the two polymorphic variants of FcγRIIIa (F^158^ and V^158^; Fig. 3C; Supplementary Fig. S5). Expression of FcγRIIIa and FcγRIIa polymorphic variants (V^158^ and H^131^) has been associated with an increased overall response rate and PFS in HER2+ patients treated with trastuzumab. This is postulated to be due to higher affinity to the IgG1 Fc region and thus increased ability to perform effector functions such as ADCC (14). Antibody modification to increase affinity to the lower-affinity allelic variant FcγRIIIa-F^158^ may expand the population of patients responsive to antibody therapies.

Both IgG1-GM and IgG1-DE showed increased affinity for both allelic variants of FcγRIIIa. However, IgG1-DE/GM had the highest affinity for both FcγRIIIa allelic variants, with a 30-fold increase compared with IgG1-WT, a 15- to 20-fold increase compared with IgG1-GM and margetuximab, and a 5-fold increase compared with IgG1-DE (Fig. 3C). FcγRIIIa is classified as a low-to-moderate affinity receptor for IgG1. This means that monomeric IgG1 is unable to bind well and requires increased avidity, that is, the formation of an immune complex, for effective binding, retention on the immune cell surface, and immune cell activation (31). We measured the affinity of IgG1-DE/GM to both FcγRIIIa allelic variants to be higher than that to the high-affinity FcγRI. This indicates that IgG1-DE/GM constitutes a ligand of very high affinity for FcγRIIIa. IgG1-DE/GM also demonstrated increased affinity for the inhibitory receptor FcγRIIb. However, the affinity increase was moderate (3-fold), and thus, this Fc-engineered antibody features a greatly increased activating/inhibitory ratio. Furthermore, no differences between any of the anti-HER2 or the anti-FRα antibody variants were observed for binding to FcRn at either pH 7.4 or pH 6.0 (Supplementary Fig. S6).

**Figure 4.**
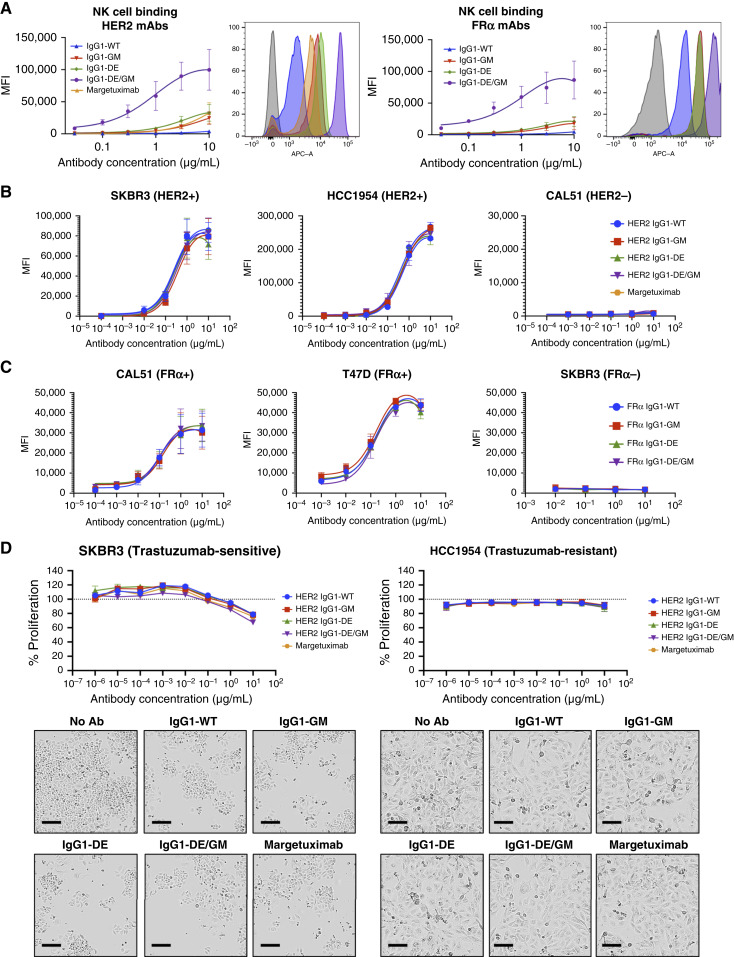
Cell binding and Fab-mediated functionality of FcγRIIIa-enhanced antibodies. **A,** Binding of anti-HER2 and anti-FRα IgG variants (0.03–10 µg/mL) to NK cells evaluated by flow cytometry (*n* = 4). **B **and** C,** Binding of monomeric anti-HER2 IgG variants (0.0001–10 µg/mL) to HER2-expressing (SKBR3 and HCC1954) or nonexpressing (CAL51) cells (*n* = 3–6; **B**) or to FRα-expressing (CAL51 and T47D) or nonexpressing (SKBR3) cells (*n* = 3–6; **C**). **D,** Impact of antibodies on proliferation detected by live-cell imaging (Incucyte; *n* = 3). Representative images of cells treated with 10 μg/mL anti-HER2 antibody variants for 5 days. Scale bar, 100 μm. MFI, median fluorescence intensity.

Human NK cells express only FcγRIIIa (Fig. 1C and D), and monomeric anti-HER2 and anti-FRα IgG1-WT display very poor binding to NK cells (Fig. 4A and B). IgG1-GM and IgG1-DE bound monomerically, although weakly, to FcγRIIIa on NK cells. However, consistent with SPR data (Fig. 3C), IgG1-DE/GM demonstrated strong monomeric binding to FcγRIIIa on NK cells, approximately 100-fold greater K_D_ than IgG1-WT (Fig. 4A).

Fc modification did not affect Fab-mediated antigen recognition (Fig. 4B, HER2; Fig. 4C, FRα) among all variants. Similarly, we measured no differences in the Fab-mediated proliferation inhibition of trastuzumab-sensitive SKBR3 cells, whereas no variants showed any direct inhibition against trastuzumab-resistant HCC1954 cells (Fig. 4D).

In summary, combining glycoengineering and DE point mutations greatly enhances antibody affinity for both FcγRIIIa polymorphic variants while retaining the Fab-mediated specific tumor antigen recognition, binding characteristics, and antiproliferative effects of the original clone.

### Fc-engineered antibodies, especially IgG1-DE/GM, engender enhanced NK cell activation and subsequent reduced FcγRIIIa expression

To evaluate if IgG1-DE/GM’s enhanced affinity for FcγRIIIa translated into NK cell stimulation, we assessed degranulation, calcium flux, ADCC, and cytokine release.

**Figure 5.**
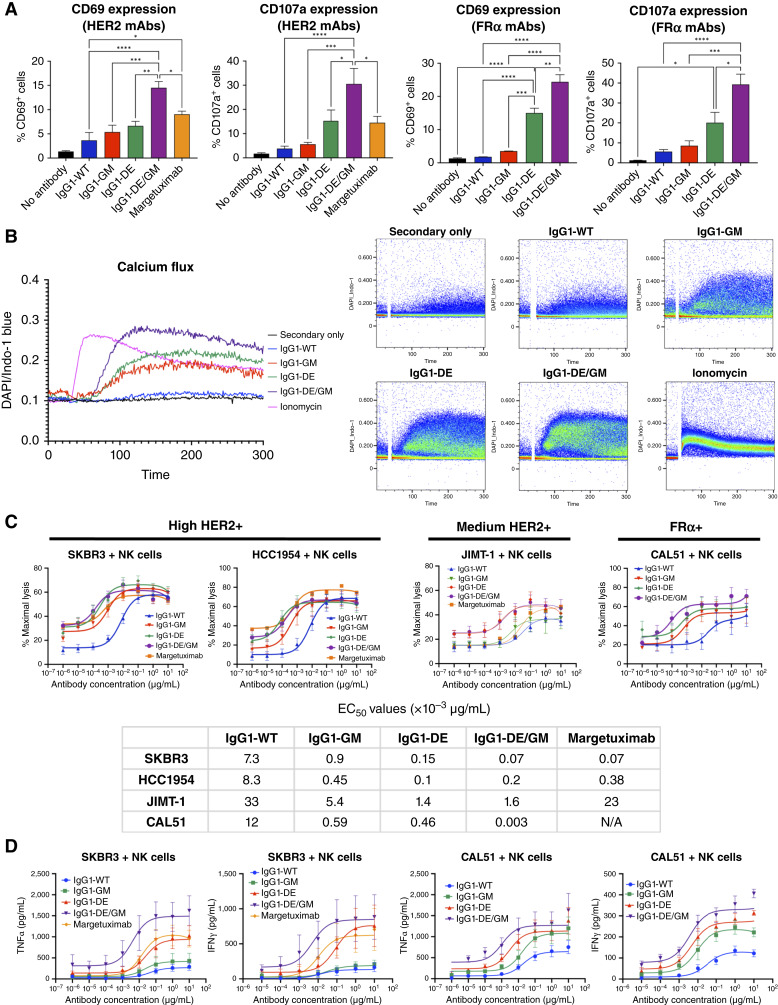
FcγRIIIa-enhanced antibodies engender NK cell activation above IgG1-WT. **A,** CD69 and CD107a expression on NK cells measured by flow cytometry following cross-linking of FcγRIIIa by engineered antibodies (20 μg/mL) and anti-F(ab′)_2_ cross-linker (10 μg/mL; *n* = 3–4). Statistical significance was determined using one-way ANOVA. **B,** Intracellular calcium flux of purified human NK cells after cross-linking antibody variants with an anti-IgG. Ionomycin was used as a positive control. **C,** ADCC measured by LDH release in a 4-hour coculture of target cells (SKBR3, HCC1954, JIMT-1, CAL51) and purified NK cells (1:10) in the presence of antibodies (0.000001-10 μg/mL; *n* = 7–12). **D,** TNFα and IFNγ release measured by ELISA from a 6-hour coculture of target cells and purified NK cells (1:10) in the presence of antibodies (0.000001-10 μg/mL; *n* = 5). *, *P* < 0.05; **, *P* < 0.01; ***, *P* < 0.001; ****, *P* < 0.0001.

CD107a (or LAMP-1) transiently appears on the NK cell surface during degranulation, indicating functional activity, whereas upregulated CD69 marks FcγRIIIa-mediated NK cell activation. For anti-HER2 antibodies, IgG1-DE/GM engendered significantly increased CD69 expression and CD107a expression compared with IgG1-WT, IgG1-GM, IgG1-DE, and the clinically used Fc-enhanced margetuximab (Fig. 5A). Similarly, for anti-FRα antibodies, CD69 and CD107a expression induced by IgG1-DE/GM was significantly increased compared with other variants.

We measured calcium flux as a marker of NK cell intracellular activation following cross-linking of the antibodies. Ionomycin was used as a non-FcR–mediated positive control. Consistent with enhanced FcγRIIIa-mediated immune cell stimulation and degranulation, IgG1-DE/GM induced the greatest calcium flux of all variants, and IgG1-DE and IgG1-GM showed increased calcium flux over IgG1-WT (Fig. 5B).

We measured NK cell–mediated ADCC of cancer cells at 10:1 and 3:1 effector/target ratios. At the 10:1 ratio, IgG1-GM, IgG1-DE, and IgG1-DE/GM, as well as margetuximab, engendered higher ADCC by NK cells against HCC1954 and SKBR3, demonstrating an approximate 40- and 100-fold decrease in EC_50_, respectively, for IgG1-DE/GM compared with IgG1-WT (Fig. 5C). In the 3:1 ratio using the high HER2-expressing SKBR3, IgG1-DE and IgG1-DE/GM had superior ADCC at a low concentration (0.1 ng/mL) compared with all other variants, including the Fc-enhanced margetuximab (Supplementary Fig. S7). For the medium/low HER2-expressing cancer cell line JIMT-1, IgG1-DE and IgG1-DE/GM had greater ADCC compared with the other variants in the 10:1 model (Fig. 5C), whereas IgG1-DE/GM trended highest in the 3:1 model (Supplementary Fig. S7). Contrastingly, anti-FRα IgG1-DE/GM triggered the most ADCC against CAL51 cells, with a ∼100-fold decrease in EC_50_ compared with IgG1-GM and IgG1-DE and a ∼4,000-fold decrease compared with IgG1-WT (Fig. 5C).

After 6-hour coculture, IgG1-DE/GM was the most potent TNFα and IFNγ inducer at all antibody concentrations (Fig. 5D). Anti-HER2 IgG1-DE and margetuximab also significantly increased cytokine release over IgG1-WT and IgG1-GM. Similarly, anti-FRα IgG1-DE/GM induced the highest levels of cytokines although IgG1-GM also triggered more release than IgG1-WT.

**Figure 6.**
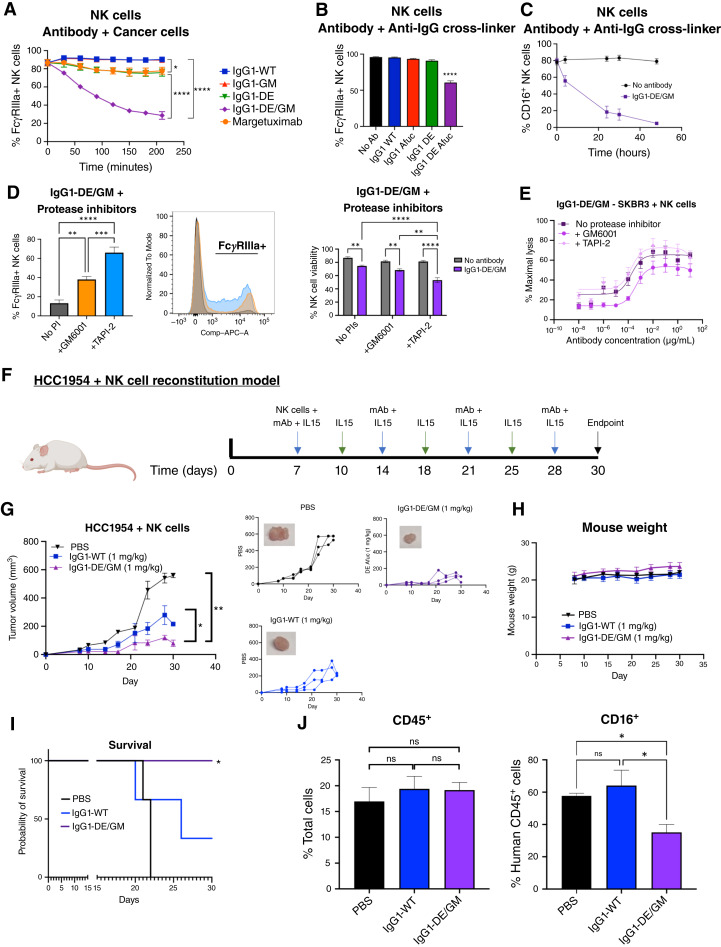
Loss of FcγRIIIa expression after antibody engagement on NK cells, but FcγRIIIa-enhanced antibody reduces xenograft growth compared with IgG1-WT. **A,** Expression of FcγRIIIa was measured by flow cytometry following coculture of cancer cells and purified NK cells (1:10), depicting the effects of cross-linking in an antigen-specific manner by multiple copies of the target antigen expressed on the surface of tumor cells (*n* = 3). **B,** FcγRIIIa expression on NK cells after cross-linking engineered antibodies in an antigen-independent manner with anti-Fab polyclonal antibodies, in the absence of cancer cells, for 4 hours (*n* = 3). **C,** FcγRIIIa expression on NK cells after cross-linking IgG1-DE/GM with anti-Fab for 48 hours (*n* = 6). **D,** Expression of FcγRIIIa and DAPI+ NK cells in a coculture of target cells and purified NK cells (1:10) treated with 10 µg/mL IgG1-DE/GM and protease inhibitors (50 µmol/L; *n* = 3). **E,** ADCC measured by LDH release following a 4-hour coculture of target cells and purified NK cells (1:10) treated with antibodies and protease inhibitors (50 µmol/L; *n* = 3). **F,** Flow chart of HCC1954 xenografts reconstituted with human NK cells. **G,** Tumor growth curves of mice treated with NK cells and weekly antibody (1 mg/kg) or PBS and biweekly 250 ng IL15. **H,** Mouse weight measurements. **I,** Kaplan–Meier plot of the probability of survival of mice, with “death” occurring when the tumor reaches 250 mm^3^. **J,***Ex vivo* tumors were evaluated by flow cytometry. Left, percentage of human CD45^+^ cells out of total viable cells; right, percentage of human CD16^+^ cells out of total human CD45^+^ cells. All statistical significance was determined using one-way ANOVA or the log-rank test for the Kaplan–Meier plot. *, *P* < 0.05; **, *P* < 0.01; ***, *P* < 0.001; ****, *P* < 0.0001; ns, not significant.

Stimulation of effector cells via FcγR can reduce their expression (32, 33). To establish whether enhanced engagement of Fc-modified antibodies influenced FcγRIIIa levels on the NK cell surface, we studied FcγRIIIa expression over time after stimulation with antibody variants. IgG1-DE/GM cross-linking in an antigen-dependent manner in the presence of high HER2+ HCC1954 cells induced a rapid loss of FcγRIIIa from NK cells, whereas IgG1-GM and IgG1-DE caused moderate reductions. Little FcγR loss was seen with IgG1-WT (Fig. 6A). These results are consistent with previous reports (32, 33) and suggest that FcγRIIIa loss may correlate with increasing affinity for the receptor.

We then tested if FcγRIIIa loss was an NK cell–intrinsic mechanism by cross-linking antibodies with an anti-IgG polyclonal antibody to mimic immune complex formation on the NK cell surface in the absence of cancer cells. Only IgG1-DE/GM significantly decreased FcγRIIIa expression after 4 hours (Fig. 6B), which was sustained for 48 hours (Fig. 6C), indicating that FcγRIIIa loss is an intrinsic regulatory mechanism for NK cells and not an effect induced by the cancer cells.

FcγRIIIa can be cleaved off the surface of NK cells after activation via the protease ADAM-17 (34). Using the ADAM-17 inhibitor TAPI-2 or the MMP inhibitor GM6001 we found that incubation with these protease inhibitors did not affect NK cell viability or FcγRIIIa expression (Fig. 6D). However, FcγRIIIa expression was retained following stimulation with IgG1-DE/GM in the presence of TAPI-2, indicating that cleavage of FcγRIIIa by ADAM-17 may be a mechanism involved in the reduction of FcγRIIIa expression (Fig. 6D). However, activation of NK cells with IgG1-DE/GM in the presence of TAPI-2 resulted in decreased NK cell viability. This is likely due to NK cells becoming unable to detach from target cells. As FcγRIIIa cleavage plays a critical role in NK cell detachment, higher affinity variants prolong attachment to the target cell, leading to more activation-induced death of NK cells (33). Furthermore, we measured comparable levels of ADCC by IgG1-DE/GM in the presence or absence of TAPI-2 (Fig. 6E). This may be due to a combination of the ability of IgG1-DE/GM to trigger strong killing of cancer cells, balanced against the inability of NK cells to detach from the target cells and thus subsequently induce serial killing, alongside higher levels of NK cell death in the presence of the inhibitor and antibody (33).

To determine whether the enhanced cleavage of FcγRIIIa from NK cells by IgG1-DE/GM would impair antibody efficacy *in vivo*, we established HCC1954 tumors in NSG mice reconstituted with human NK cells and dosed weekly with a suboptimal concentration (1 mg/kg) of IgG1-WT or IgG1-DE/GM (Fig. 6F). HCC1954 is resistant to the Fab-mediated effects of trastuzumab (Fig. 4D); thus, any effects could only be attributed to the antibody–NK cell engagement and effector functions. IgG1-WT was able to partly restrict tumor growth compared with the vehicle control; however, IgG1-DE/GM induced significant tumor restriction and a probability of survival superior to IgG1-WT and the vehicle control (Fig. 6G and I). No toxicities or weight loss was observed (Fig. 6H). There was similar engraftment of human CD45^+^ cells in all three conditions (Fig. 6J). However, we observed lower FcγRIIIa expression on immune cells in xenografts treated with IgG1-DE/GM, indicating that in concordance with *in vitro* data, tumor-associated NK cells may show reduced FcγRIIIa expression following antibody treatment (Fig. 6J).

Overall, IgG1-DE/GM induced greater NK cell activation, cytokine release, and ADCC although moderate levels of ADCC may be due to protease-induced cleavage of FcγRIIIa from the NK cell surface, a potential regulatory mechanism of NK cell activation. However, IgG1-DE/GM still restricted tumor growth better than IgG1-WT, showing that NK cells can retain functional potency and superior antitumor effects *in vivo*.

### Fc-engineered antibodies potentiate a proinflammatory phenotypic shift in human macrophages

Macrophages are prominent effector cells in the TME and can be broadly classified into classically activated M1-like, able to exert proinflammatory, antitumoral effects, or alternatively activated M2-like, with immunomodulatory, protumoral attributes. We showed that macrophages express FcγRIIIa, as well as FcγRI, FcγRIIa, and the inhibitory FcγRIIb (see Fig. 1). We hypothesized that macrophage activation may be influenced by antibodies with enhanced affinity to FcγRIIIa.

**Figure 7.**
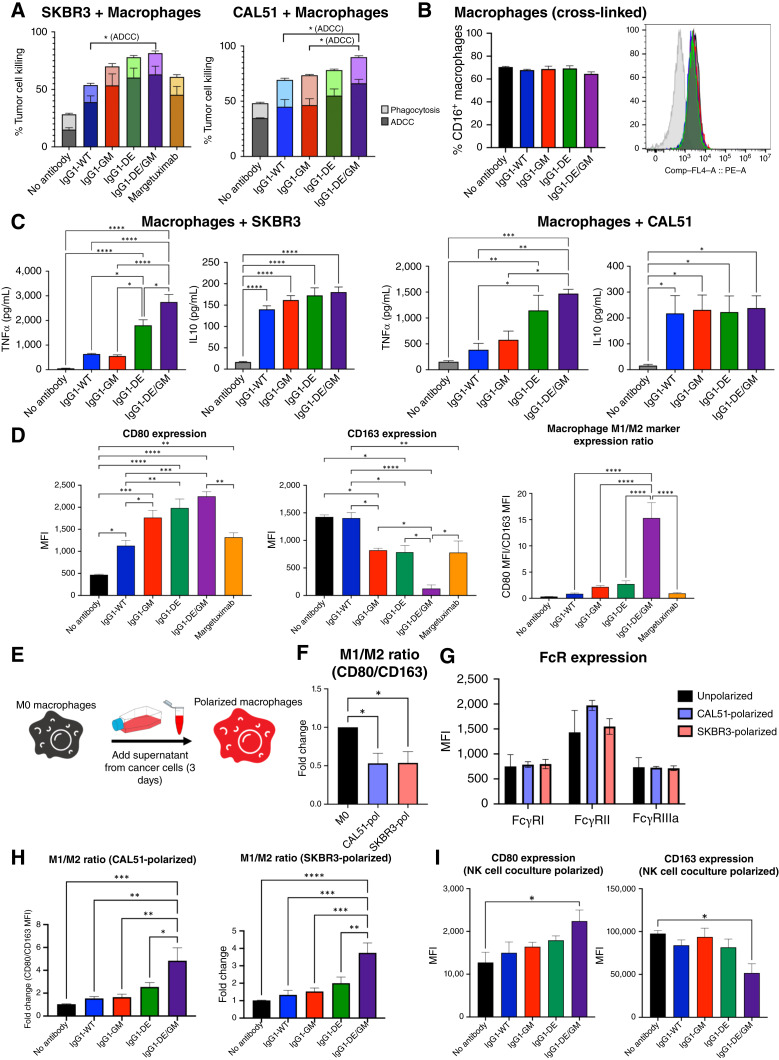
Macrophage activation by FcγRIIIa-enhanced antibodies. **A,** Fluorometric analysis of ADCC/ADCP in 3.5-hour cocultures of macrophages and target cells (3:1) in the presence or absence of antibodies (0.1 μg/mL). Target cells were labeled with CSFE, macrophages were stained with anti-CD14-APC, and cell viability was measured by DAPI (*n* = 3). **B,** CD16a expression on macrophages after 3.5 hours with antibodies (10 μg/mL) and anti-F(ab′)_2_ (5 μg/mL) or in the presence of target cells (5:1; *n* = 4). **C,** TNFα and IL10 cytokine release measured by ELISA of a 16-hour coculture of macrophages and target cells (5:1) in the presence of antibodies (10 μg/mL; *n* = 3–6). **D,** CD80 and CD163 expression by flow cytometry on macrophages after a 16-hour coculture of macrophages and target cells (5:1 ratio) in the presence of antibodies (10 μg/mL). M1/M2 ratio was calculated by dividing the CD80 median fluorescence intensity (MFI) by CD163 MFI (*n* = 3–5). **E,** Generation of cancer cell–polarized macrophages. **F,** M1/M2 ratio was calculated by dividing the CD80 MFI by CD163 MFI for macrophages polarized by CAL51 or SKBR3 supernatant (*n* = 4–6). **G,** FcγRI, FcγRII, and FcγRIIIa expression on macrophages polarized by CAL51 or SKBR3 supernatant (*n* = 4). **H,** M1/M2 ratio for macrophages cocultured with cancer cells treated with antibodies (*n* = 6–7). **I,** CD80 and CD163 expression on macrophages after a 16-hour incubation with supernatants derived from a 6-hour coculture of NK cells and cancer cells (10:1) with/without antibodies (10 μg/mL; *n* = 3). Statistical significance was determined using one-way ANOVA. *, *P* < 0.05; **, *P* < 0.01; ***, *P* < 0.001; ****, *P* < 0.0001.

We evaluated macrophages for their ability to induce ADCC/ADCP against target cells in the presence of Fc-engineered antibodies. Although all engineered antibodies trended toward higher ADCC compared with IgG1-WT, only IgG1-DE/GM had significantly increased ADCC (SKBR3: 38.9% IgG1-WT vs. 63.0% IgG1-DE/GM; CAL51: 45.0% IgG1-WT vs. 66.0% IgG1-DE/GM; Fig. 7A). No differences in ADCP were observed. One of the limiting factors of NK cell activation with IgG1-DE/GM was the reduction of FcγRIIIa from the surface of NK cells after antibody stimulation. Contrastingly, FcγRIIIa cell surface levels on macrophages remained unchanged 3.5 hours after antibody stimulation (Fig. 7B).

We next measured the secretion of the proinflammatory cytokine TNFα and the anti-inflammatory cytokine IL10 in cocultures of human macrophages and cancer cells (5:1 ratio) incubated with engineered antibodies. For anti-HER2 mAbs, IgG1-DE/GM induced the highest TNFα levels compared with other variants, whereas IgG1-DE had increased production of TNFα compared with IgG1-WT and IgG1-GM, which induced similar levels of TNFα (Fig. 7C). Contrastingly, no differences in IL10 production were measured between the variants. The superiority of IgG1-DE/GM in triggering the highest TNFα levels was recapitulated with the anti-FRα mAb panel (Fig. 7C). In concordance with the anti-HER2 mAb panel, IL10 production was also similar between all anti-FRα variants. Thus, IgG1-DE/GM was superior in inducing proinflammatory TNFα release, whereas it did not alter IL10 secretion by macrophages.

M1- and M2-like macrophages can be broadly distinguished based on CD80 and CD163, respectively. As Fc-engineered antibodies potentiated proinflammatory TNFα production and macrophages are known to be influenced by cytokines, we evaluated whether macrophage phenotype was altered by antibody variants in cocultures with cancer cells. In *ex vivo* cultures, IgG1-GM, IgG1-DE, and IgG1-DE/GM all induced upregulation of CD80 compared with IgG1-WT (1.7-, 1.9-, and 2.2-fold). IgG1-DE/GM also induced an 11-fold downregulation of CD163 compared with IgG1-WT (Fig. 7D). IgG1-DE/GM had the greatest CD80 upregulation and the greatest CD163 downregulation of all variants, also demonstrated by an enhanced M1/M2 marker ratio. These findings indicate an IgG1-DE/GM–driven macrophage transition to an M1-like, and thus more proinflammatory and more likely antitumoral, phenotype.

Macrophages in the TME can be modulated toward alternatively activated phenotypes by cancer cells. We conditioned macrophages using breast cancer cell supernatants for 3 days and then cocultured them with breast cancer cells (Fig. 7E). These cancer cell-polarized macrophages had a more immunosuppressive phenotype, characterized by a decreased M1/M2 marker ratio (Fig. 7F). However, no differences in FcγRI (CD64), FcγRII (CD32), or FcγRIIIa (CD16) expression were observed in the cancer cell–conditioned macrophages, indicating that they have the potential to respond to antibody stimulation (Fig. 7G). When activated with IgG1-DE/GM, tumor-conditioned macrophages demonstrated a significantly increased M1/M2 marker ratio compared with other conditions (Fig. 7H). These findings indicate that despite being polarized to an anti-inflammatory state, macrophages may be amenable to a proinflammatory shift upon stimulation by an FcγRIIIa-enhanced antibody.

We next sought to understand whether this macrophage phenotype shift toward a more M1-like state requires direct stimulation of macrophages or whether external factors, such as other activated cells that release cytokines, could also influence macrophage phenotype. We incubated macrophages with supernatants from cocultures of NK cells and cancer cells with Fc-engineered antibodies. Similarly to conditions in which macrophages were directly activated in cocultures with cancer cells and antibodies, supernatants from NK cells, cancer cells, and IgG1-DE/GM stimulated CD80 upregulation and CD163 downregulation on the macrophage surface compared with supernatants from other antibody variant cocultures (Fig. 7I). These findings suggest that activation of cells such as NK cells in the TME by IgG1-DE/GM may trigger the release of cytokines and other soluble factors that could alter tumor-conditioned macrophages in the TME toward a proinflammatory phenotype.

In *ex vivo* cultures, IgG1-DE/GM was the most potent in inducing ADCC and proinflammatory TNFα production, but there was no increase in anti-inflammatory IL10. Macrophages, including cancer-conditioned macrophages, were polarized toward an M1-like phenotype by IgG1-DE/GM over other variants. These data indicate that FcγRIIIa-enhanced antibodies may induce a switch in breast cancer–conditioned macrophage polarization toward a more proinflammatory state.

### FcγRIIIa-enhanced antibodies display superior *in vivo* efficacy compared with WT equivalent antibodies in HER2+ and TNBC models

FcγRIIIa-enhanced engineered antibodies potently induce NK cell activation and a breast cancer–conditioned macrophage proinflammatory shift. We established xenografts in immunocompromised NSG mice, deficient in B, T, and NK cells, allowing for the introduction of human immune cells (5).

**Figure 8.**
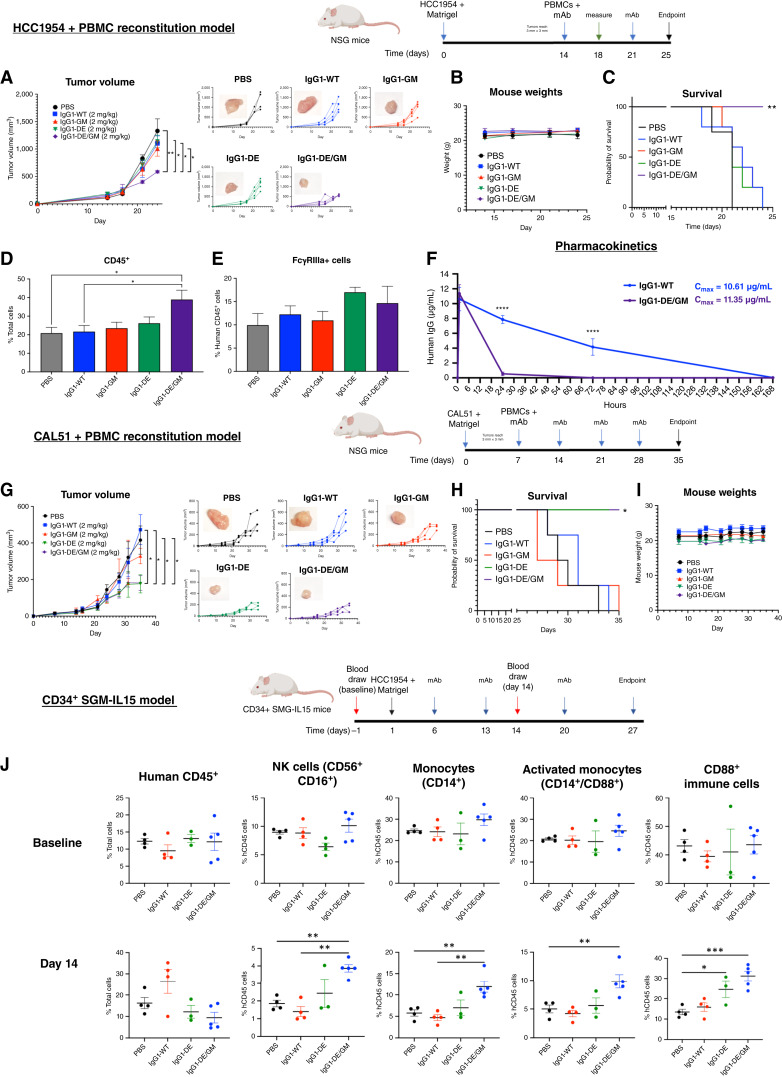
*In vivo* effects of Fc-enhanced IgG1 antibodies. **A,** HCC1954 growth curves in mice injected with 10^7^ PBMCs and treated weekly with 2 mg/kg antibody or PBS. **B,** Mouse weight measurement. **C,** Kaplan–Meier plot of the probability of survival of mice, with “death” occurring when the tumor reached 250 mm^3^. Statistical significance was determined using the log-rank test. **D** and **E,** Human CD45^+^ (**D**) and CD16^+^ (**E**) cells extracted from HCC1954 tumors at endpoint, evaluated by flow cytometry. **F,** Serum clearance analysis of antibodies (*n* = 5 per time point). Statistical analysis was determined using the Šidák multiple comparisons test. **G,** CAL51 growth curves in mice injected with 10^7^ PBMCs and treated weekly with 2 mg/kg antibody or PBS. Statistical significance was determined using one-way ANOVA. **H,** Kaplan–Meier plot of the probability of survival of mice, with “death” occurring when the tumor reached 750 mm^3^. **I,** Mouse weight measurement. **J,** Human immune cell frequencies in the serum of CD34^+^ NOD.Cg-*Prkdc*^scid^ Il2^rgtm1Wjl^ Tg(CMV-IL3,CSF2,KITLG)1Eav Tg(IL15) (NSG-SGM3-IL15) mice with HCC1954 tumors at days 0 and 14 after weekly antibody (3 mg/kg) or PBS treatment. *, *P* < 0.05; **, *P* < 0.01; ***, *P* < 0.001; ****, *P* < 0.0001.

HCC1954 xenografts are resistant to Fab-mediated antiproliferative effects of trastuzumab; thus, therapeutic efficacy would be via Fc-mediated functions. In an HCC1954 orthotopic model reconstituted with human PBMCs, mice were treated weekly with suboptimal (2 mg/kg) antibody doses of trastuzumab variants. Tumor growth in IgG1-WT–treated mice was comparable with PBS control, IgG1-GM, and IgG1-DE. However, IgG1-DE/GM significantly reduced tumor growth (Fig. 8A), had no impact on mouse weight (Fig. 8B) or signs of overt toxicity, and increased the probability of survival (Fig. 8C). Furthermore, tumors treated with IgG1-DE/GM had increased human CD45^+^ (Fig. 8D) and CD16^+^ (FcγRIIIa) immune cell infiltrates (Fig. 8E). Interestingly, when injected into nontumor–bearing NSG mice, the DE/GM showed significantly faster clearance from the circulation (>90% cleared within 24 hours) compared with WT (Fig. 8F) despite similar C_max_ values initially (10.61 μg/mL for IgG1-WT vs. 11.35 μg/mL for IgG1-DE/GM).

Similarly to the HCC1954 model, anti-FRα IgG1-DE/GM significantly restricted CAL51 xenografts and increased the probability of survival compared with IgG1-WT and PBS (Fig. 8G and H). Unlike in the HER2+ model, both IgG1-DE and IgG1-DE/GM showed comparable ability to restrict FRα+ tumor growth. No decrease in weight or toxicities was observed in the mice (Fig. 8I).

To determine how our Fc-enhanced antibodies influence immune cells in the blood, a CD34^+^ cell engrafted model (SGM3-IL15) was employed in which HCC1954 tumors were established in the mammary fat pad, and then weekly intravenous treatment with a high concentration of antibody (3 mg/kg) was performed. Although no differences in circulating human immune cell populations were observed before treatment (baseline), IgG1-DE/GM–treated mice, but not mice treated with IgG1-WT or IgG1-DE, showed significant increases in circulating NK cells (CD56^+^CD16^+^), monocytes (CD14^+^), and activated monocytes (CD14^+^CD88^+^/CD88^+^) levels compared with no antibody controls (Fig. 8J). These findings are consistent with *in vitro* data supporting superior effector functions of IgG1-DE/GM in activating NK cells and myeloid cells compared with IgG1-WT or other Fc-engineered variants. In this study, a higher dose of antibody was used weekly (3 mg/kg), and under these conditions, IgG1-WT, IgG1-DE, and IgG1-DE/GM all effectively prolonged survival, whereas none of the variants had any significant effects on mouse weights (Supplementary Fig. S8). This may indicate that Fc-engineered variants may be better utilized at doses that are suboptimal for the corresponding WT antibodies, as observed in the other models described in this study (Fig. 8A and G).

Thus, IgG1-DE/GM targeting two breast cancer–associated antigens, HER2 and FRα, delivered at suboptimal concentrations demonstrated superior tumor growth restriction, prolonged survival of mice, induced immune cell infiltration in tumors, and sustained circulating NK cell and activated monocyte levels compared with the IgG1-WT counterparts *in vivo*, despite faster clearance of IgG1-DE/GM.

## Discussion

Enhancing antibody immune stimulatory functions for cancer therapy has historically been applied in settings agnostic of the immunologic conditions required for antibodies and immune cells to function effectively in the TME. Guided by assessing the presence of FcγR-expressing immune effector cells in the breast cancer TME, including NAC- and immunotherapy-resistant disease, we designed and evaluated Fc-engineered antibodies with enhanced affinity for the prominently expressed FcγRIIIa.

Although previous studies have demonstrated the beneficial effects of afucosylation or S^239^D/I^332^E individually in trastuzumab (30, 35), our study is the first to combine glycoengineering and direct point mutation strategies into the same antibody structure to generate WT and Fc-engineered antibodies targeting two breast cancer–associated antigens, HER2 and the emerging TNBC target FRα (5). Fc-enhanced antibodies stimulate effector cells to produce proinflammatory cytokines, skew tumor-conditioned macrophages toward proinflammatory states, and promote immune effector mechanisms against HER2+ and TNBC cells. Fc-engineered antibodies restrict xenograft growth at doses suboptimal for the corresponding WT antibodies and recruit FcγRIIIa-expressing cells into the TME of orthotopically grown xenografts (5).

Removal of the core fucose from the N-linked glycan selectively improves FcγRIIIa binding due to steric inhibition caused by fucose on conventional antibodies. This has been successfully implemented in seven FDA-approved antibodies for cancer and immune-mediated disorders (data obtained from The Antibody Society: https://www.antibodysociety.org/antibody-therapeutics-product-data/). Our glycoengineered antibodies not only were completely afucosylated but also demonstrated high mannose content, as well as loss of sialic acid and galactose residues. In addition to the loss of fucose, these altered glycan profiles may influence antibody activity. The presence of sialic acid residues has been postulated to improve ADCC/CDC activity (36), whereas mannose content has been linked to serum clearance (37). In nontumor-bearing mice, IgG1-DE/GM demonstrates significantly reduced serum persistence compared with IgG1-WT (Fig. 8I) despite no differences in FcRn binding (Supplementary Fig. S6). Increased serum clearance is therefore likely due to non-FcRn–mediated recycling mechanisms, for example, the higher mannose content of the N-linked glycan in the IgG1-DE/GM variant, which we also report in our study (Fig. 3B). Despite faster clearance from the serum within 24 hours, IgG1-DE/GM is still able to engender potent antitumoral effects *in vivo*. These findings present new opportunities to potentially enhance these antitumoral functions through further engineering and glycoengineering to fine-tune the antibody’s glycans. For instance, it may be possible to reduce mannose content in order to extend the antibody half-life while retaining an afucosylated structure to support strong effector functions. Although uncoupling the contribution of each glycan in complex molecules such as antibodies is challenging, our study points to significantly enhanced affinity and effector functions of glycomodified antibodies targeting HER2+ and FRα+ breast cancers. Our data also provide novel insights into the functional attributes of Fc-engineered antibodies to guide further optimization in the future.

Point mutations introduced in IgG1 Fc can also alter affinity to FcγRs. Notably, margetuximab was designed with identical variable regions to trastuzumab but with five mutated amino acids. This agent has demonstrated increased PFS compared with trastuzumab (13). Thus, enhancing FcγRIIIa affinity may be a strategy to reduce patient variation in response to IgG1 treatments such as trastuzumab, which is known to recruit and stimulate immune effector cells via Fc to induce antitumor effects. Our FcγRIIIa-enhanced antibody significantly impairs HCC1954 tumor growth; therefore, enhanced effector functions might also provide an opportunity to treat trastuzumab-resistant patients.

Immune cells in some patients carry the lower-affinity FcγRIIIa polymorphic variant, FcγRIIIa-F^158^, and these patients are reported to experience lower response rates to trastuzumab treatment (26), compared with those with the higher-affinity polymorphic variant FcγRIIIa-V^158^. The combined FcγRIIIa-enhancing point mutations and glycomodifications in the same antibody structure greatly increase affinity for both polymorphic alleles of FcγRIIIa, which, as with margetuximab, should eliminate the differences in patient responses to conventional antibody therapy. We show the affinity of IgG1-DE/GM for FcγRIIIa to be elevated to levels higher than that of IgG1-WT for the high-affinity receptor FcγRI. This increase in affinity, beyond that previously reported for currently clinically used antibodies for cancer therapy, eliminates the need for the formation of a high-avidity immune complex otherwise needed for a WT IgG1 to robustly bind to FcγR and to be retained on immune effector cells sufficiently to engender prolonged immune surveillance. Thus, this Fc-enhanced antibody may be able to be retained in the absence of target antigen and to bind and activate immune cells at lower doses. Supporting this premise, we observe enhanced NK cell activation and xenograft growth restriction at doses in which the corresponding IgG1-WT, including trastuzumab, has little effect (Figs. 5 and 6). This may be beneficial for situations both in circulation and in the TME in which large immune complexes cannot be formed, that is, in circulating tumor cells and in micrometastases in which few target-expressing cancer cells are available.

When tested at higher doses, no differences in therapeutic activity between the variants were observed (Supplementary Fig. S8). At these concentrations, IgG1-WT is also very effective at restricting tumor growth. It is possible that the high affinity of Fc-engineered antibodies may translate to optimal performance at lower concentrations at which the WT antibodies are ineffective, potentially reducing on-target, off-tumor side effects such as trastuzumab-induced cardiotoxicity. Our bioinformatics analyses also demonstrate the variability of patient expression of FcRs in tumors, made more complicated by the polymorphic variants of FcγRIIIa, which influence antibody efficacy. This variability in FcR expression and polymorphisms, and thus how the Fc-enhanced antibodies may perform in a real clinical setting, cannot be fully assessed in mouse models. However, our data indicate that there may be advantages to the use of Fc engineered antibodies, particularly for subsets of patients with the FcγRIIIa-F^158^ polymorphic variant who do not respond well to WT trastuzumab or for patients with high FcγRIIIa expression in tumors, and this can only be further explored in clinical testing.

Furthermore, in CD34-engrafted mice, treatment with the anti-HER2 IgG1-DE/GM, but not with anti-HER2 IgG1-DE or anti-HER2 IgG1-WT, was associated with significantly higher levels of NK cells, monocytes, and activated monocytes in circulation after 14 days compared with vehicle and IgG1-WT controls. These findings, consistent with enriched immune cell infiltration in PBMC-engrafted models, further support the ability of IgG1-DE/GM to engage and stimulate tumor-resident and peripheral immune effector cells.

Treatment with IgG1-DE/GM *in vitro* results in the reversal of alternatively activated immune effector cell features and the promotion of proinflammatory effector cell polarization. Furthermore, IgG1-DE/GM induces greater macrophage-mediated ADCC and repolarizes tumor-conditioned macrophages toward a more proinflammatory, antitumoral phenotype (Fig. 7). Additionally, *in vivo*, we report evidence of retention or enhancement of monocyte and stimulated monocyte levels in circulation and in immune cell recruitment into tumors. This may improve therapeutic function not only toward the primary tumor but also by harnessing antibody-loaded immune cells in early metastatic sites, such as lung metastases, in which macrophages are detected (38) and in which these cells may support metastatic initiation and tumor progression (39, 40). It is therefore possible that IgG1-DE/GM may potentiate broader immune-activating effects that extend beyond the immediate targeting of antigen-expressing tumor cells.

We show that FcγRIIIa-expressing macrophages are retained in residual TNBC (Fig. 2). This patient group has previously been shown to have PD-1/PD-L1–low tumors and shows enrichment for an exhausted NK cell phenotype (7). Thus, targeting the residual tumor-associated macrophages through an Fc-engineered antibody may reeducate these cells toward a proinflammatory phenotype and consequently influence proinflammatory cytokines to enhance antitumor responses.

One mechanism NK cells employ to prevent overstimulation is FcγRIIIa shedding by the release of the protease inhibitor ADAM-17 following activation (34). The degree of shedding correlates with the increase in antibody affinity for FcγRIIIa (Fig. 6). This can be inhibited by protease inhibitors such as TAPI-2 and GM-6001; however, no further increase in ADCC is observed. This is likely due to the requirement of FcγRIIIa shedding for NK cells to be able to detach from cancer cells and perform serial killing, a mechanism that has been previously reported (33). Although the loss of FcγRIIIa is observed to be sustained over several days (Fig. 6), NK cells alone are able to restrict xenograft growth over the course of at least a month (Fig. 6), indicating that FcγRIIIa loss may be transient. Furthermore, the increased release of proinflammatory cytokines by NK cells following IgG1-DE/GM engagement (Fig. 5) may enhance antitumor effects through other mechanisms. For example, IFNγ release by NK cells can prime macrophage activation and promote proinflammatory cytokine secretion and phagocytosis (41). NK cell–produced TNFα and IFNγ induce ICAM-1 upregulation on K562 cancer cells, which then enhances the cytotoxicity of cancer cells by promoting their conjugate formation with NK cells (42). Additional studies are required to explore the mechanisms by which NK cells restore Fc receptor expression and cytotoxic attributes over time.

Our FcγRIIIa-enhanced antibody was designed based on robust FcγRIIIa expression on NK cells and myeloid cells in tumor lesions and the correlation of expression with the therapeutic efficacy of trastuzumab (Fig. 1). These findings support the notion of (i) tailored treatment approaches based on the characterization of potential effector cells in the TME of resistant tumors and (ii) an approach that evaluates the conditions required and the antibody engineering necessary to achieve successful responses in specific cancer subtypes and potentially individual patients. However, as subsets of breast cancers do not express FcγRIIIa or indeed any FcγR in the TME (Fig. 1), such patients may require other strategies, such as antibody–drug conjugates, which do not require immune cell engagement.

In summary, informed by the expression and distribution of FcγRIIIa and FcγR-expressing cells in therapy-resistant and aggressive breast cancer subsets, we combined antibody glycoengineering and point mutation to develop antibodies with greatly enhanced affinity to FcγRIIIa, allowing engagement with immune cells without the requirement of immune complex formation. This can result in heightened immune surveillance and anticancer effects by immune cells, including those from individuals with the low-affinity FcγRIIIa variant who would not otherwise benefit from conventional antibody therapies such as trastuzumab. Fc-engineered antibodies potentiate enhanced NK cell activation, polarize tumor-conditioned macrophages toward classically activated antitumoral phenotypes, and skew the immune cytokine environment to a proinflammatory state. Fc-enhanced antibodies exert antitumor functions *in vivo* at suboptimal doses, which are insufficient for conventional and current clinically available antibodies to exert tumor-restricting effects. The Fc-enhanced anti-HER2 antibody shows greater efficacy against trastuzumab-resistant disease *in vivo*. Potentiating the effector functions of tumor-infiltrating FcγRIIIa-expressing immune cells may be a promising tailored strategy that could be matched to the appropriate TME conditions of patients with aggressive and treatment-resistant diseases.

## Supplementary Material

Supplementary Figure 1 Supplementary Figure 1: Bulk RNA analysis of the Guy’s Cohort of primary TNBC patients and stratification of patients based of FcγR expression.

Supplementary Figure 2 Supplementary Figure 2: Schema of antibody PIPE cloning and production of antibody variants.

Supplementary Figure 3 Supplementary Figure 3: Non-reduced and reduced SDS-PAGE gels of antibody variants demonstrating structural integrity.

Supplementary Figure 4 Supplementary Figure 4: Comparison of commercial trastuzumab with the equivalent anti-HER2 IgG1-WT made in-house. HER2-expressing (SKBR3 breast cancer cells were incubated on ice with antibody variants (0.0001–10µg/mL) for 30 minutes and detected using anti-F(ab’)2-Alexa Fluor 647 (n = 3, mean ± SEM). NK cells were incubated on ice with antibody variants (0.03–10µg/mL) for 30 minutes and detected using anti-F(ab’)2-Alexa Fluor 647 (n = 3, mean ± SEM). ADCC measured by LDH release in a 4-hour co-culture of target cells and purified NK cells (1:10 ratio) in the presence of antibodies (10-0.000001 µg/mL).

Supplementary Figure 5 Supplementary Figure 5: SPR analysis curves of antibody variants to each FcγR. His-tagged FcγR were captured by immobilized His tag antibodies on the surface of the SPR chip, and antibody variants were flowed over the FcγR-bound surface over a concentration range of 1 to 1000nM. Curves shown are at a concentration of 1 to 100nM (FcγRI and FcγRIIIa) or 10 to 1000nM (FcγRIIa and FcγRIIb).

Supplementary Figure 6 Supplementary Figure 6: Binding of antibody variants to FcRn at pH 7.4 and pH 6.0. Recombinant human HER2 or FRα was coupled to microbeads and incubated with antibody variants for 2 hours, before being stained with PE-labelled tetramerized recombinant FcRn at either pH 7.4 or pH 6.0 and analyzed by flow cytometry. Data is n = 2-4, mean ± SEM.

Supplementary Figure 7 Supplementary Figure 7: ADCC measured by flow cytometric analysis using Zombie Green Fixable Viability dye in a 4-hour co-culture of target cells and purified NK cells (1:3 ratio) in the presence of engineered antibodies (0.1 ng/mL). Bars represent n = 2 independent experiments with two different human NK cell donors, % specific lysis ± SEM.

Supplementary Figure 8 Supplementary Figure 8: Effects of antibodies in tumor bearing mice engrafted with human CD34+ cells. (Left) Kaplan-Meier plot of probability of survival of mice (death occurring when tumor reached 15 × 15mm). Statistical significance was determined using a Log-Rank test compared to PBS control. (Right) Mouse weights measured during treatment.

Supplementary Table 1 Supplementary Table 1: Patient characteristics of bioinformatics analyses

Supplementary Table 2 Supplementary Table 2: PIPE PCR primers and cycling conditions.

Supplementary Table 3 Supplementary Table 3: Antibody variant production yield from transient transfection of 30mL Expi293F cells.

## Data Availability

Publicly available data reanalyzed in this study were obtained from the Gene Expression Omnibus: GSE76360, GSE109710, GSE176078, GSE169246, and GSE210616. All other raw data generated in this study are available from the corresponding author upon request.
