# Supplementary material for: An Fc-Engineered Glycomodified Antibody Supports Proinflammatory Activation of Immune Effector Cells and Restricts Progression of Breast Cancer
Source: Cancer Res. 2025 Oct 23;85(22):4521–40. doi: 10.1158/0008-5472.CAN-24-3174 (PMC12616241; doi:10.1158/0008-5472.CAN-24-3174)
Supplement: Supplementary Figure 1 — Bulk RNA analysis of the Guy’s Cohort of primary TNBC patients and stratification of patients based of FcγR expression. [file can-24-3174_supplementary_figure_1_suppsf1.docx]

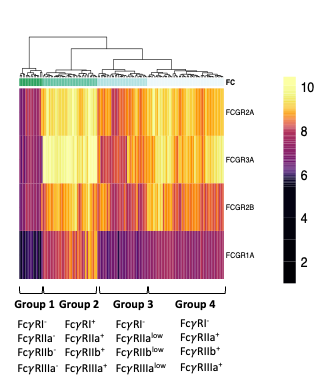


**Supplementary Figure 1:** Bulk RNA analysis of the Guy’s Cohort of primary TNBC patients and stratification of patients based of FcγR expression.
