# Supplementary material for: An Fc-Engineered Glycomodified Antibody Supports Proinflammatory Activation of Immune Effector Cells and Restricts Progression of Breast Cancer
Source: Cancer Res. 2025 Oct 23;85(22):4521–40. doi: 10.1158/0008-5472.CAN-24-3174 (PMC12616241; doi:10.1158/0008-5472.CAN-24-3174)
Supplement: Supplementary Figure 2 — Schema of antibody PIPE cloning and production of antibody variants. [file can-24-3174_supplementary_figure_2_suppsf2.docx]

**Supplementary Figure 2:** Schema of antibody PIPE cloning and production of antibody variants.
