# Supplementary material for: An Fc-Engineered Glycomodified Antibody Supports Proinflammatory Activation of Immune Effector Cells and Restricts Progression of Breast Cancer
Source: Cancer Res. 2025 Oct 23;85(22):4521–40. doi: 10.1158/0008-5472.CAN-24-3174 (PMC12616241; doi:10.1158/0008-5472.CAN-24-3174)
Supplement: Supplementary Figure 3 — Non-reduced and reduced SDS-PAGE gels of antibody variants demonstrating structural integrity. [file can-24-3174_supplementary_figure_3_suppsf3.docx]

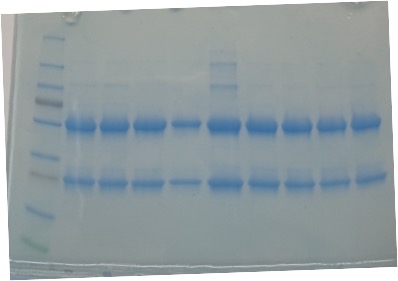

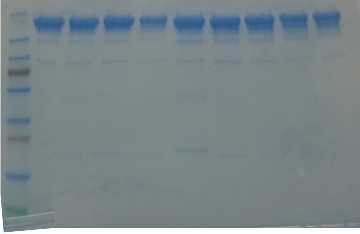


**Anti-HER2**

**WT**

**GM**

**DE**

**DE/**

**GM**

**Marg**

**WT**

**GM**

**DE**

**DE/**

**GM**

**Anti-FRα**

**Anti-HER2**

**WT**

**GM**

**DE**

**DE/**

**GM**

**Marg**

**WT**

**GM**

**DE**

**DE/**

**GM**

**Anti-FRα**

**(kDa)**

**250**

**130**

**100**

**70**

**55**

**35**

**25**

**15**

**10**

**(kDa)**

**250**

**130**

**100**

**70**

**55**

**35**

**25**

**15**

**10**

**Supplementary Figure 3:** Non-reduced and reduced SDS-PAGE gels of antibody variants demonstrating structural integrity.
