# Supplementary material for: An Fc-Engineered Glycomodified Antibody Supports Proinflammatory Activation of Immune Effector Cells and Restricts Progression of Breast Cancer
Source: Cancer Res. 2025 Oct 23;85(22):4521–40. doi: 10.1158/0008-5472.CAN-24-3174 (PMC12616241; doi:10.1158/0008-5472.CAN-24-3174)
Supplement: Supplementary Figure 4 — Comparison of commercial trastuzumab with the equivalent anti-HER2 IgG1-WT made in-house. HER2-expressing (SKBR3 breast cancer cells were incubated on ice with antibody variants (0.0001–10µg/mL) for 30 minutes and detected using anti-F(ab’)2-Alexa Fluor 647 (n = 3, mean ± SEM). NK cells were incubated on ice with antibody variants (0.03–10µg/mL) for 30 minutes and detected using anti-F(ab’)2-Alexa Fluor 647 (n = 3, mean ± SEM). ADCC measured by LDH release in a 4-hour co-culture of target cells and purified NK cells (1:10 ratio) in the presence of antibodies (10-0.000001 µg/mL). [file can-24-3174_supplementary_figure_4_suppsf4.docx]

**Supplementary Figure 4:** Comparison of commercial trastuzumab with the equivalent anti-HER2 IgG1-WT made in-house. HER2-expressing (SKBR3 breast cancer cells were incubated on ice with antibody variants (0.0001-10µg/ml) for 30 minutes and detected using anti-F(ab’)_2_-Alexa Fluor 647 (n=3, mean +/- SEM). NK cells were incubated on ice with antibody variants (0.03-10µg/ml) for 30 minutes and detected using anti-F(ab’)_2_-Alexa Fluor 647 (n=3, mean +/- SEM). ADCC measured by LDH release in a 4-hour co-culture of target cells and purified NK cells (1:10 ratio) in the presence of antibodies (10-0.000001 μg/ml).
