# Supplementary material for: An Fc-Engineered Glycomodified Antibody Supports Proinflammatory Activation of Immune Effector Cells and Restricts Progression of Breast Cancer
Source: Cancer Res. 2025 Oct 23;85(22):4521–40. doi: 10.1158/0008-5472.CAN-24-3174 (PMC12616241; doi:10.1158/0008-5472.CAN-24-3174)
Supplement: Supplementary Figure 5 — SPR analysis curves of antibody variants to each FcγR. His-tagged FcγR were captured by immobilized His tag antibodies on the surface of the SPR chip, and antibody variants were flowed over the FcγR-bound surface over a concentration range of 1 to 1000nM. Curves shown are at a concentration of 1 to 100nM (FcγRI and FcγRIIIa) or 10 to 1000nM (FcγRIIa and FcγRIIb). [file can-24-3174_supplementary_figure_5_suppsf5.docx]

**Supplementary Figure 5:** SPR analysis curves of antibody variants to each FcγR. His-tagged FcγR were captured by immobilized His tag antibodies on the surface of the SPR chip, and antibody variants were flowed over the FcγR-bound surface over a concentration range of 1-1000nM. Curves shown are at a concentration of 1-100nM (FcγRI and FcγRIIIa) or 10-1000nM (FcγRIIa and FcγRIIb).
