# Supplementary material for: An Fc-Engineered Glycomodified Antibody Supports Proinflammatory Activation of Immune Effector Cells and Restricts Progression of Breast Cancer
Source: Cancer Res. 2025 Oct 23;85(22):4521–40. doi: 10.1158/0008-5472.CAN-24-3174 (PMC12616241; doi:10.1158/0008-5472.CAN-24-3174)
Supplement: Supplementary Figure 6 — Binding of antibody variants to FcRn at pH 7.4 and pH 6.0. Recombinant human HER2 or FRα was coupled to microbeads and incubated with antibody variants for 2 hours, before being stained with PE-labelled tetramerized recombinant FcRn at either pH 7.4 or pH 6.0 and analyzed by flow cytometry. Data is n = 2-4, mean ± SEM. [file can-24-3174_supplementary_figure_6_suppsf6.docx]

**Supplementary Figure 6:** Binding of antibody variants to FcRn at pH 7.4 and pH 6.0. Recombinant human HER2 or FRα was coupled to microbeads and incubated with antibody variants for 2 hours, before being stained with PE-labelled tetramerized recombinant FcRn at either pH 7.4 or pH 6.0 and analyzed by flow cytometry. Data is n=2-4, mean +/- SEM.
