# Supplementary material for: An Fc-Engineered Glycomodified Antibody Supports Proinflammatory Activation of Immune Effector Cells and Restricts Progression of Breast Cancer
Source: Cancer Res. 2025 Oct 23;85(22):4521–40. doi: 10.1158/0008-5472.CAN-24-3174 (PMC12616241; doi:10.1158/0008-5472.CAN-24-3174)
Supplement: Supplementary Figure 7 — ADCC measured by flow cytometric analysis using Zombie Green Fixable Viability dye in a 4-hour co-culture of target cells and purified NK cells (1:3 ratio) in the presence of engineered antibodies (0.1 ng/mL). Bars represent n = 2 independent experiments with two different human NK cell donors, % specific lysis ± SEM. [file can-24-3174_supplementary_figure_7_suppsf7.docx]

**Supplementary Figure 7**: ADCC measured by flow cytometric analysis using Zombie Green Fixable Viability dye in a 4-hour co-culture of target cells and purified NK cells (1:3 ratio) in the presence of engineered antibodies (0.1 ng/ml). Bars represent n=2 independent experiments with two different human NK cell donors, % specific lysis +/- SEM.
