# Supplementary material for: An Fc-Engineered Glycomodified Antibody Supports Proinflammatory Activation of Immune Effector Cells and Restricts Progression of Breast Cancer
Source: Cancer Res. 2025 Oct 23;85(22):4521–40. doi: 10.1158/0008-5472.CAN-24-3174 (PMC12616241; doi:10.1158/0008-5472.CAN-24-3174)
Supplement: Supplementary Figure 8 — Effects of antibodies in tumor bearing mice engrafted with human CD34+ cells. (Left) Kaplan-Meier plot of probability of survival of mice (death occurring when tumor reached 15 × 15mm). Statistical significance was determined using a Log-Rank test compared to PBS control. (Right) Mouse weights measured during treatment. [file can-24-3174_supplementary_figure_8_suppsf8.docx]

**Supplementary Figure 8:** Effects of antibodies in tumor bearing mice engrafted with human CD34+ cells**.** (Left) Kaplan-Meier plot of probability of survival of mice (death occurring when tumor reached 15x15mm). Statistical significance was determined using a Log-Rank test compared to PBS control. (Right) Mouse weights measured during treatment.
