# Supplementary material for: An Fc-Engineered Glycomodified Antibody Supports Proinflammatory Activation of Immune Effector Cells and Restricts Progression of Breast Cancer
Source: Cancer Res. 2025 Oct 23;85(22):4521–40. doi: 10.1158/0008-5472.CAN-24-3174 (PMC12616241; doi:10.1158/0008-5472.CAN-24-3174)
Supplement: Supplementary Table 1 — Patient characteristics of bioinformatics analyses [file can-24-3174_supplementary_table_1_suppst1.docx]

**Supplementary Table 1:** Patient characteristics of bioinformatics analyses

| **Cohort** | **Samples** | **Figure** |
| --- | --- | --- |
| GSE76360 (Bulk) | 48 primary HER2+ sampled before trastuzumab treatment | Figure 1e |
| GSE109710 (Bulk) | 173 primary HER2+ sampled before trastuzumab treatment | Figure 1f |
| Guy’s Cohort (Bulk) | 140 primary TNBC | Supplementary Figure 1 |
| KCH Cohort (NanoString) | 9 TNBC (samples taken pre- and post-NAC) | Figure 2d |
| NKI Cohort (Nanostring) | 12 TNBC (samples taken pre- and post-NAC) | Figure 2d |
| GSE176078 (scRNA) | 3 primary HER2+, 4 primary TNBC | Figure 1a, b, c, d |
| GSE169246 (scRNA) | 5 matched primary TNBC pre- and post-chemotherapy | Figure 2a, b, c |
| Bassez Cohort (scRNA) | 12 primary TNBC (pre-treatment), 6 primary TNBC (post-PD-1 therapy) | Figure 2e, f |
| GSE210616 (Spatial transcriptomic) | 24 pre-treatment samples from 12 TNBC patients, 17 samples post-NAC from 9 TNBC patients | Figure 1g, h, and Figure 2g, h, i |
