# Supplementary material for: An Fc-Engineered Glycomodified Antibody Supports Proinflammatory Activation of Immune Effector Cells and Restricts Progression of Breast Cancer
Source: Cancer Res. 2025 Oct 23;85(22):4521–40. doi: 10.1158/0008-5472.CAN-24-3174 (PMC12616241; doi:10.1158/0008-5472.CAN-24-3174)
Supplement: Supplementary Table 2 — PIPE PCR primers and cycling conditions. [file can-24-3174_supplementary_table_2_suppst2.docx]

**Supplementary Table 2:** PIPE PCR primers and cycling conditions.

| **Primer Name** | **Sequence** |
| --- | --- |
| F-S239D | CAGCACCTGAACTCCTGGGGGGACCGGACGTCTTCCTCTTCCCCC |
| R-S239D | GGGGGAAGAGGAAGACGTCCGGTCCCCCCAGGAGTTCAGGTGCTG |
| F-I332E | CCAGCCCCCGAAGAGAAAACCATCTCCAAAGCCAAAGGGCAGCCCC |
| R-I332E | GGGGCTGCCCTTTGGCTTTGGAGATGGTTTTCTCTTCGGGGGCTGG |
| F-Uni1 | CATTGGGTTTCTGCTGCTCTGGGTTCCAGCTAGCCGCGGT |
| R-Uni1 | ACCGCGGCTAGCTGGAACCCAGAGCAGCAGAAACCCAATG |
| F-Uni2 | GTTGCTTTGATTACAACACTGGAGAGAAATGCCAGCATGTTGCTGATT |
| R-Uni2 | GGGGGAAGAGGAAGACGTCCGGTCCCCCCAGGAGTTCAGGTGCTG |

| **Step** | **Temperature** | **Duration** | **Number of Cycles** |
| --- | --- | --- | --- |
| Initial Denaturation | 98°C | 30 seconds | 1 |
| Denaturation | 98°C | 10 seconds | 30 |
| Annealing | 60°C | 15 seconds | 30 |
| Extension | 72°C | (15 x 1kb) seconds | 30 |
| Store | 4°C | ∞ | 1 |
