# Supplementary material for: An Fc-Engineered Glycomodified Antibody Supports Proinflammatory Activation of Immune Effector Cells and Restricts Progression of Breast Cancer
Source: Cancer Res. 2025 Oct 23;85(22):4521–40. doi: 10.1158/0008-5472.CAN-24-3174 (PMC12616241; doi:10.1158/0008-5472.CAN-24-3174)
Supplement: Supplementary Table 3 — Antibody variant production yield from transient transfection of 30mL Expi293F cells. [file can-24-3174_supplementary_table_3_suppst3.docx]

**Supplementary Table 3:** Antibody variant production yield from transient transfection of 30mL Expi293F cells.

| **Antibody** | **Production (g/L)** |
| --- | --- |
| Anti-HER2 IgG1-WT | 1.13 ± 0.2 |
| Anti-HER2 IgG1-GM | 1.21 ± 0.17 |
| Anti-HER2 IgG1-DE | 1.22 ± 0.42 |
| Anti-HER2 IgG1-DE/GM | 1.11 ± 0.22 |
| Anti-FRα IgG1-WT | 1.31 ± 0.11 |
| Anti-FRα IgG1-GM | 1.32 ± 0.16 |
| Anti-FRα IgG1-DE | 1.23 ± 0.33 |
| Anti-FRα IgG1-DE/GM | 1.09 ± 0.25 |
